# Multifunctional Bio-Gels in Environmental Remediation: Current Advances and Future Perspectives

**DOI:** 10.3390/gels11110864

**Published:** 2025-10-28

**Authors:** Baolei Liu, Shixing Zhang, Lingfeng Zhao, Cunyou Zou, Jianlong Xiu

**Affiliations:** 1 State Key Laboratory of Low Carbon Catalysis and Carbon Dioxide Utilization (Yangtze University), Wuhan 430100, China; 2 Key Laboratory of Exploration Technologies for Oil and Gas Resources (Yangtze University), Ministry of Education, Wuhan 430100, China; 3 School of Petroleum Engineering, Yangtze University, Wuhan 430100, China; zsx_parallel@163.com (S.Z.); 2025730027@yangtzeu.edu.cn (L.Z.); 4 PetroChina Research Institute of Petroleum Exploration & Development, Beijing 100083, China; zoucunyou@petrochina.com.cn (C.Z.); xiujianlong69@petrochina.com.cn (J.X.)

**Keywords:** bio-gel, environmental remediation, heavy metal removal, stimuli-responsive gel, ecological restoration, biodegradability

## Abstract

Bio-gels are a class of functional polymeric materials with three-dimensional network structures. Their exceptional biocompatibility, biodegradability, high specific surface area, and tunable physicochemical properties make them highly promising for environmental remediation. This article systematically reviews the classification of bio-gels based on source, cross-linking mechanisms, and functional attributes. It also elaborates on their fundamental properties such as porous structure, high water absorbency, stimuli-responsiveness, and mechanical stability and examines how these properties influence their environmental remediation efficiency. This review comprehensively analyze the mechanisms and efficacy of bio-gels in adsorbing heavy metal ions, removing organic dyes, improving soil water retention, and restoring ecosystems. Special attention is given to the interactions between surface functional groups and contaminants, the role of porous structures in mass transfer, and the ecological effects within soil–plant systems. Additionally, this review explores extended applications of bio-gels in medical tissue engineering, controlled release of drugs and fertilizers, and enhanced oil recovery, highlighting their versatility as multifunctional materials. Finally, based on current progress and challenges, this review outline key future research directions. These include elucidating microscopic interaction mechanisms, developing low-cost renewable feedstocks, designing multi-stimuli-responsive structures, improving long-term stability, and establishing full life-cycle environmental safety assessments. These efforts will help advance the efficient, precise, and sustainable use of bio-gels in environmental remediation, offering innovative solutions to complex environmental problems.

## 1. Introduction

The ongoing process of global industrialization has led to increasingly severe environmental issues, including heavy metal contamination [1,2], soil desertification, drought [3,4], ecosystem degradation, and emissions of persistent organic pollutants [5,6,7]. For example, heavy metal pollution is evident in aquatic systems, where crustaceans accumulate metals beyond safe levels due to their benthic feeding habits. In some coastal regions of China, copper and cadmium concentrations in crabs have reached 528 mg·kg^−1^ and 24 mg·kg^−1^, respectively, significantly exceeding international safety standards [2]. Meanwhile, soil desertification and drought are projected to affect up to 6 billion people facing water scarcity by 2050 [3]. Furthermore, Fusarium head blight, caused by Fusarium species, affects over 20% of China’s wheat cultivation area annually, resulting in yield losses of 3.4 million tons [7]. These challenges pose serious threats to ecological security and human health, representing major obstacles to sustainable development [6,8,9,10].

Although conventional remediation methods such as physical adsorption, chemical precipitation, and biodegradation [11] remain in use, they often suffer from limited efficiency, high operational costs, and risks of secondary pollution. Therefore, developing environmentally friendly, efficient, and sustainable remediation materials and technologies has become a crucial research direction. In this context, bio-gels have emerged as a promising class of bio-based functional materials with significant potential in environmental remediation. Bio-gels are polymeric materials featuring three-dimensional network structures [12,13], and they have attracted growing attention in environmental applications [14]. Typically derived from natural polymers such as polysaccharides and proteins, bio-gels offer wide availability, excellent biocompatibility, and biodegradability [15]. Their structural properties provide abundant surface functional groups (e.g., hydroxyl, carboxyl, and amino), which enable effective contaminant binding and tunable physicochemical behavior. In practical applications, bio-gels exhibit high water absorbency and retention, along with a large specific surface area that facilitates pollutant adsorption and immobilization [16,17]. Studies indicate that bio-gels can effectively remove heavy metal ions through coordination, ion exchange, and electrostatic interactions. They also adsorb and degrade organic pollutants such as dyes, antibiotics, and pesticides via hydrophobic interactions and hydrogen bonding. In soil remediation, bio-gels help improve soil structure, enhance water retention [3,4], regulate nutrient release, and provide favorable microenvironments for functional microorganisms [12,18], thereby supporting ecosystem restoration.

Despite the promising prospects of bio-gels in environmental remediation, several challenges hinder their practical engineering applications. First, under varying environmental conditions such as extreme pH, high salinity, or complex microbial systems bio-gels often exhibit insufficient mechanical strength and long-term stability, which directly affects their service life and remediation efficiency [19,20]. Second, the diversity of raw material sources and variability in preparation methods result in performance fluctuations between batches, complicating large-scale implementation [21]. Additionally, key aspects such as adsorption selectivity for specific contaminants, material reusability, and post-use disposal strategies require further optimization. Of particular concern is the need to systematically assess the environmental degradation products of bio-gels and their potential ecotoxicity.

**Figure 1:**
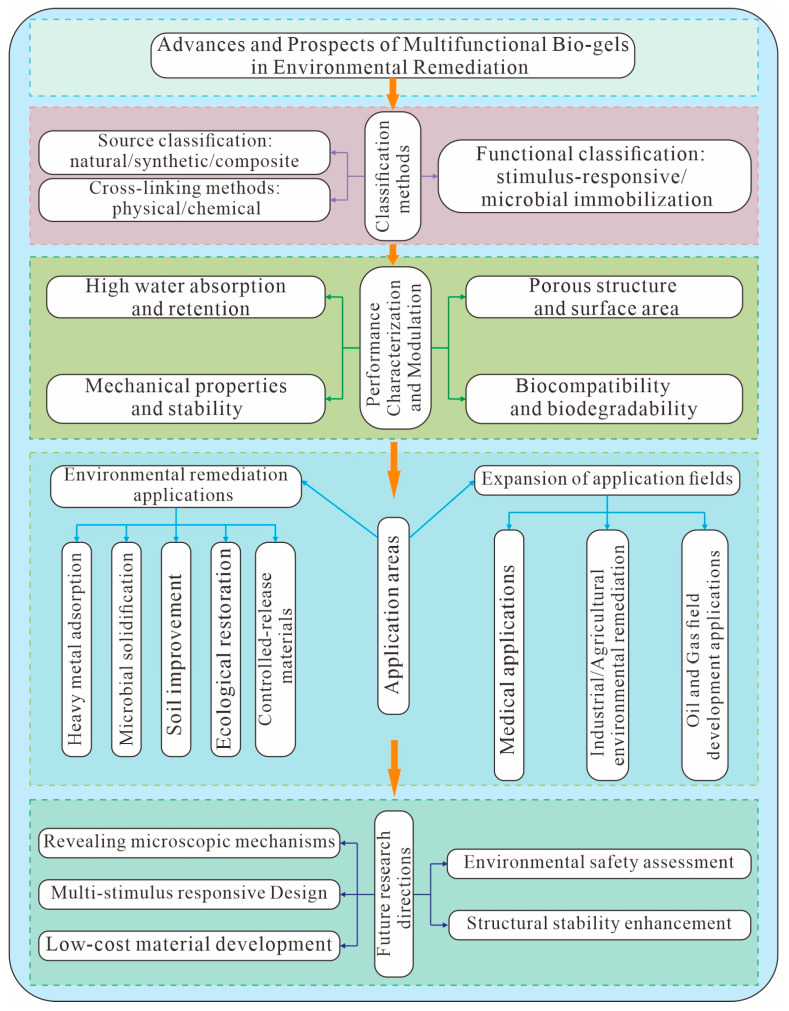
Flowchart of research progress on bio-gels.

In light of the current research status, this article systematically reviews the classification, structural characteristics, and key mechanisms of bio-gels in environmental remediation. It specifically examines recent advances in heavy metal adsorption, organic pollutant degradation, soil improvement, and microbial immobilization mediated by bio-gels. The influence of material porosity, water retention capacity, stimuli-responsive properties, and environmental stability on remediation efficiency is thoroughly analyzed. Furthermore, this work outlines future research directions, including molecular design, structure activity relationships, and environmental behavior and safety assessments of bio-gels. The aim is to provide a theoretical foundation and technical support for advancing the precise and large-scale application of bio-gels in environmental remediation. The research framework and main content of this study are summarized in Figure 1.

## 2. Classification Criteria for Bio-Gels

Bio-gels can be classified using a multi-dimensional framework that considers their source, cross-linking mechanisms, and functional characteristics. This framework enables the precise design and selection of materials for specific environmental remediation scenarios. Furthermore, bio-gels possess notable biocompatibility, biodegradability, and modifiability, underscoring their significant application potential in this field.

### 2.1. By Source

This classification approach is the most fundamental and widely used, as it determines the gel’s basic biocompatibility and environmental friendliness.

(1) Natural bio-gels: Derived from animal, plant, or microbial sources, these gels exhibit excellent biocompatibility, complete biodegradability, and low environmental toxicity. Polysaccharide-based gels, such as those composed of chitosan and alginate, are particularly effective biopolymers for heavy metal adsorption. In chitosan, the amino (-NH_2_) and hydroxyl (-OH) groups on its molecular chains act as coordination sites, enabling effective binding with various heavy metal ions, including Zn^2+^, Pb^2+^, Co^2+^, and Cd^2+^. Consequently, cross-linked chitosan gel demonstrates high adsorption capacity for these ions [16]. Sodium alginate gels are also widely applied, showing high adsorption capacity, rapid kinetics, and favorable reusability in heavy metal removal [12]. Protein-based gels, such as those derived from gelatin and collagen, serve dual roles in environmental remediation as heavy metal adsorbents [17] and microbial immobilization carriers [13]. They not only effectively remove heavy metals and dyes via their functional groups but also provide a favorable microenvironment for degrading microorganisms, enabling more complex and sustained bioaugmentation.
(2) Composite bio-gels: To overcome the limitations of single-component materials, composite bio-gels are developed by blending natural and synthetic polymers. For instance, hydrogels fabricated from chitosan and acrylamide integrate the efficient adsorption properties of the natural biopolymer with the superior mechanical strength and stability of the synthetic polymer, thereby mitigating the drawbacks of each individual component [22]. In another approach, anionic and cationic gels synthesized by copolymerizing N-isopropylacrylamide (NIPAM) with acrylic acid or chitosan, respectively, were mixed together. This mixture minimized intra-particle association and resulted in an enhanced overall adsorption capacity [23]. Furthermore, ternary composite hydrogel beads prepared from sodium alginate (SA), cellulose nanofibers (CNF), and polyethyleneimine (PEI) demonstrated highly efficient Cr(VI) removal from aqueous solutions, retaining 95.69% of their initial adsorption capacity after five adsorption–desorption cycles [24]. Composite bio-gels thus enable precise design of structures and functionalities for more complex, efficient, and intelligent applications.
(3) Synthetic bio-gels: These gels are artificially synthesized via chemical polymerization of materials such as polyacrylamide (PAM), polyvinyl alcohol (PVA), and polyethylene glycol (PEG). Synthetic hydrogels containing functional groups like amino groups (-NH_2_) can effectively complex and remove metal ions from solutions. Their advantages include a controllable structure and high stability. Furthermore, when compounded with other substances, they can exhibit significantly enhanced mechanical strength and generate synergistic complexation effects. Within such composite networks, these systems can achieve a synergistic adsorption outcome where the combined effect exceeds the sum of individual contributions (“1 + 1 > 2” effect) [25].

### 2.2. By Cross-Linking Mechanism

The cross-linking method determines the stability and responsive behavior of the gel network [26].

Physically cross-linked gels form reversible three-dimensional networks through non-covalent interactions, such as hydrogen bonding, ionic bonds, hydrophobic interactions, and host–guest interactions. The cross-linking process is mild and environmentally friendly, as it avoids residual chemical cross-linkers, thereby eliminating associated toxicity risks and preserving biocompatibility. These characteristics make physically cross-linked gels well-suited for applications requiring rapid adsorption kinetics, regenerability, and cost-effectiveness [21].

Chemically cross-linked gels possess permanent, irreversible three-dimensional networks formed by covalent bonds, typically introduced using cross-linkers or initiators to drive polymerization. This method results in superior mechanical strength, high porosity, a large specific surface area, and precisely controllable swelling behavior [15].

### 2.3. By Functional Characteristics

Stimuli-responsive smart gels undergo significant changes in volume, structure, or osmotic pressure in response to minor variations in environmental conditions such as pH, temperature, light, electric fields, or specific ions. This responsive behavior is particularly valuable for the controlled release of remediation agents (e.g., fertilizers, pesticides, microorganisms) and for enabling repeated adsorption desorption cycles [27]. Such gels also find application in heavy metal adsorption; for example, certain temperature-sensitive gels develop larger and more porous structures upon heating, leading to a notable increase in their adsorption capacity for heavy metal ions [23].

Microbial immobilization gels are designed to encapsulate microorganisms within a polymer hydrogel network, helping to maintain their viability and metabolic activity over extended biological processes. The immobilization of living cells is achieved through adhesion to the hydrogel matrix, which serves as a supportive scaffold. Key structural parameters including mesh size, porosity, hydration behavior in various colloidal environments, and interactions with organic or inorganic compounds can be modulated to regulate microbial growth and function [13].

## 3. Properties and Functions of Bio-Gels

**Figure 2:**
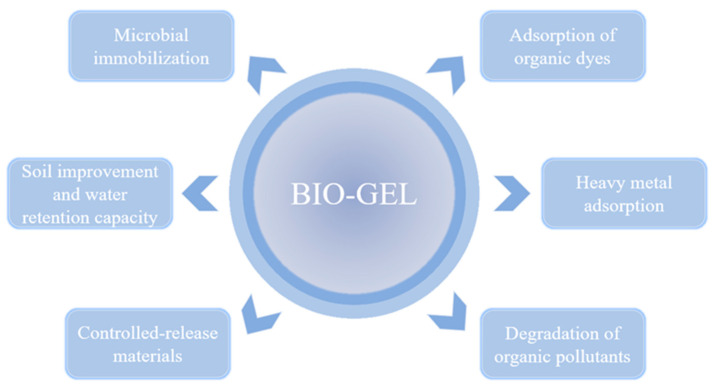
Mechanisms of bio-gels in environmental remediation.

Owing to their unique three-dimensional network structure, tunable physicochemical properties, and excellent biocompatibility, bio-gels have emerged as promising materials in environmental remediation. This section systematically reviews the mechanisms and recent advances in the use of bio-gels for heavy metal removal, microbial immobilization, soil improvement, and controlled-release applications. The primary application mechanisms are illustrated in Figure 2.

### 3.1. Properties of Bio-Gels

#### 3.1.1. Advances in Porous Structure Regulation

**Figure 3:**
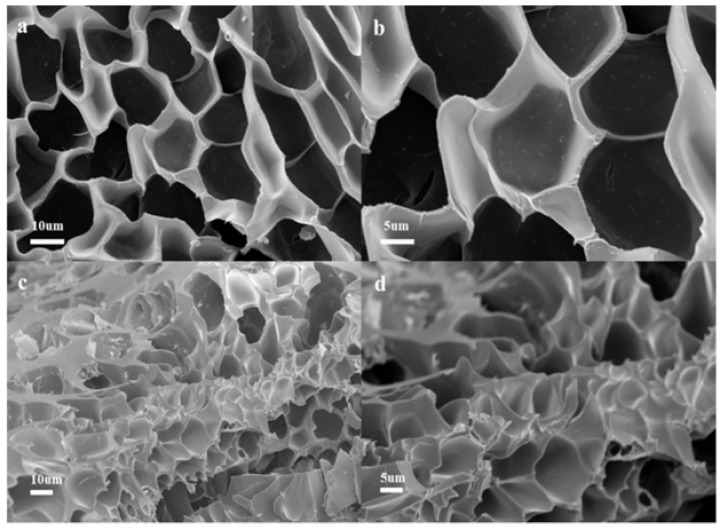
SEM images of hydrogel before (**a**,**b**) and after (**c**,**d**) adsorption [28].

The interpenetrating three-dimensional porous network and the resulting high specific surface area are defining physical characteristics of bio-gels, distinguishing them from conventional remediation materials. These structural features directly govern their efficiency in mass transfer, pollutant adsorption, and microbial immobilization. Early research primarily focused on confirming the fundamental role of this porous structure and establishing its correlation with performance. Initial investigations, using techniques such as scanning electron microscopy (SEM), directly revealed the presence of three-dimensional porous networks within the gels. For example, Wu et al. [28] clearly observed via SEM that a modified sodium alginate/polyacrylic acid (OSM/PAA) hydrogel developed a well-defined and dense porous morphology after swelling (Figure 3a,b). This structure was shown to significantly facilitate the diffusion of heavy metal ions into the gel interior, thereby enhancing its adsorption capacity. The study further compared the gel morphology after Cu(II) adsorption, revealing obvious pore filling and a reduction in pore size (Figure 3c,d). This provided direct evidence that the porous structure serves as the physical foundation for the exposure of and reaction with pollutant adsorption sites. Similarly, Petre et al. [13] highlighted the importance of the porous structure as an ideal microenvironment for microbial growth. They noted that the interconnected internal channels provide sheltered spaces and reactive sites for immobilized bacterial and fungal cells, ensuring their activity and efficiency during long-term biodegradation processes.

As research has advanced, the focus has shifted toward precise control of pore architecture to optimize gel performance. Regulating key structural parameters such as pore size, porosity, and interconnectivity during synthesis has thus become essential. Ningrum et al. [23] systematically examined the effect of synthesis temperature on the morphology of NIPAM-chitosan/NIPAM-acrylic acid hybrid gels. They observed that increasing the temperature from 10 °C to 70 °C significantly enlarged the average pore diameter, with particularly coarse and interconnected porous structures forming at 70 °C. This tailored porosity directly influenced adsorption performance: the hybrid gel achieved a Pb^2+^ adsorption capacity of 93.15 mg/g, substantially exceeding that of single-component gels (equivalent to an increase from ~0.15 to 0.30 mmol/g, nearly doubling the capacity). Adsorption selectivity followed the order Pb^2+^ > Fe^2+^ > Ni^2+^, consistent with the Hofmeister series. Furthermore, the gel exhibited a higher settled bed volume at low temperature (10 °C), which favored the exposure of adsorption sites. In contrast, at temperatures above 32 °C, hydrophobic contraction occurred, promoting pollutant desorption and material regeneration. This reversible behavior underscores the potential of such gels for repeated use. These findings demonstrate that rational pore structure design via synthesis temperature adjustment can significantly enhance both adsorption capacity and ion selectivity. This approach not only improves performance but also supports cyclic application in practical water treatment, marking an important stride toward high-performance, application-ready bio-gels.

Current research frontiers have shifted from viewing porous structures merely as static physical scaffolds toward engineering them as dynamic, multifunctional reactive platforms. By employing chemical cross-linking combined with porogen techniques, it is possible to precisely control the pore size distribution and overall porosity of bio-gels. For instance, polyacrylic acid hydrogels synthesized using novel cross-linkers allyl sorbitol (AS), allyl mannitol (AM), and allyl pentaerythritol (AP) develop a high specific surface area network. This architecture not only provides ample physical adsorption space but also incorporates abundant carboxyl groups, which effectively remove Cu(II) and Ni(II) ions via complexation and electrostatic interactions. Reported Cu(II) removal rates reached 81.8% (AS), 81.6% (AM), and 86.8% (AP), corresponding to adsorption capacities of 409, 408, and 434 mg/g, respectively. For Ni(II), removal rates were even higher: 96.8% (AS), 96.6% (AM), and 99.0% (AP), with adsorption capacities of 484, 483, and 495 mg/g. Moreover, these hydrogels exhibited strong performance in real graywater treatment, achieving up to 78% chemical oxygen demand (COD) removal, 95% total dissolved solids (TDS) removal, and a pollutant uptake capacity of 475.8 mg COD/g gel. These results underscore their practical potential for environmental applications [15].

Common porogens such as salt particles, silica microspheres, and degradable polymer microspheres are incorporated during gel synthesis and subsequently leached out or degraded after gel formation to create tailored porous architectures. Building on this approach, Wu et al. [28] grafted 2-aminopyridine onto oxidized sodium alginate via a Schiff base reaction, successfully introducing nitrogen-containing functional groups into the gel network. This modification not only preserved the high specific surface area of the porous structure but also significantly enhanced selective adsorption of heavy metal ions through the incorporation of specific coordination sites. The resulting OSM/PAA hydrogel exhibited maximum adsorption capacities of 367.64 mg/g for Cu(II), 398.4 mg/g for Zn(II), and 409.83 mg/g for Ni(II) under optimized conditions (pH 5, adsorbent dosage 0.4 g/L). Adsorption performance was superior in sulfate medium, followed by the Freundlich isotherm and pseudo-second-order kinetics, and was identified as a spontaneous, heterogeneous chemical process. Notably, the hydrogel maintained over 50% of its initial adsorption capacity after ten adsorption desorption cycles, demonstrating excellent reusability. This evolution marks a strategic shift in bio-gel research from merely optimizing physical structures toward precisely engineering chemical functionalities, thereby achieving synergistic alignment between structural design and performance.

#### 3.1.2. Advances in High Water Absorption/Retention Capacity

**Figure 4:**
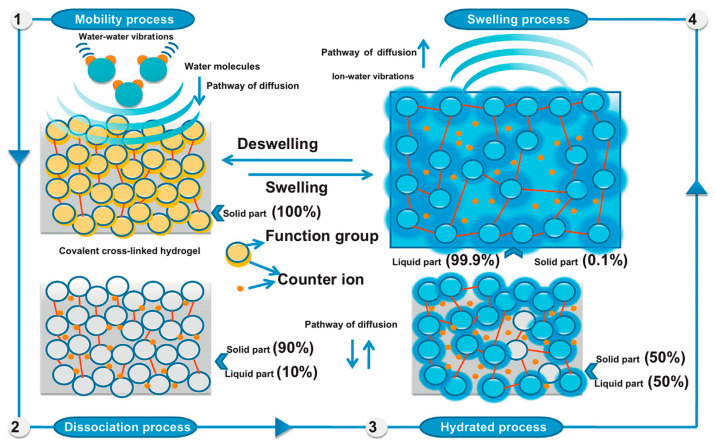
The swelling mechanism of hydrogels [3].

Considerable progress has been made in elucidating the water absorption and retention properties of bio-gels. Early research primarily centered on synthetic hydrogels, which, despite demonstrating high swelling capacity (Figure 4), often exhibited drawbacks such as poor biocompatibility and lack of biodegradability. In recent years, the research focus has shifted toward the use of natural biopolymers or agricultural waste to develop bio-based hydrogels. This transition not only aligns with green chemistry principles but also facilitates the resource utilization of waste materials. Such a paradigm shift has accelerated advances in material design and performance optimization, with the main evolutionary trends reflected in the following aspects.

In the initial stage of raw material exploration and performance evaluation, studies have demonstrated that the swelling properties of hydrogels are strongly influenced by the chemical composition of the biomass feedstocks used. For instance, Xie et al. [5] systematically compared hydrogels derived from Chinese cabbage waste (CW) and banana peel (BP). Under the lowest cross-linking density, the CW-based hydrogel achieved an equilibrium swelling ratio of 616.6 g/g in ultrapure water, significantly higher than that of the BP-based hydrogel (495.0 g/g). With a fixed monomer ratio, CW-gel-4 exhibited a swelling capacity of 551.8 g/g and maintained a water retention rate of 57.6% after drying, considerably surpassing the BP-based gel. In soil water retention tests, the CW-gel increased the water-holding capacity of sandy soil by 78.2% (from 137.2% to 244.4%) and extended the water retention duration by five days. In contrast, the BP-gel enhanced the water retention of clay soil by 43.3% (from 155.0% to 222.6%), highlighting its adaptability to different soil types. In a related study, Fang et al. [4] reported that a melon peel-based hydrogel (MP-gel), specifically the MP-gel-2 formulation, exhibited a remarkable swelling capacity of 765.6 g/g, substantially higher than the 421.4 g/g observed for an orange peel-based hydrogel (OP-gel). Under pH-responsive conditions, MP-gel-2 reached its optimal swelling ratio of 606.76 g/g at pH 8.14, while OP-gel-2 achieved 421.37 g/g at pH 8.11, indicating the superior water absorption of MP-gel under weakly alkaline conditions. Moreover, after seven reuse cycles, MP-gel retained a swelling capacity of 84.0 g/g, demonstrating excellent stability. In practical soil applications, MP-gel increased the water retention capacity of sandy soil from 137% to 271%. At addition rates of 1.5% to 2.0%, it significantly promoted the growth of wheat seedlings under drought stress, improving both plant height and biomass compared to other treatments. These comparative studies not only quantify the differences in water absorption, retention, pH adaptability, and reusability of hydrogels derived from various raw materials but also underscore that feedstock selection is crucial for developing high-performance hydrogels. This insight provides a scientific basis for the targeted screening and modification of natural polymeric materials.

Secondly, at the stage of structural regulation and mechanistic investigation, research has evolved from macroscopic performance evaluation toward uncovering the relationships between microstructural architecture and water absorption retention mechanisms. Scanning electron microscopy has been instrumental in this regard, revealing that high-performance hydrogels exhibit more abundant and interconnected porous network structures on their surfaces [3,4]. Such architecture substantially increases the contact area with water molecules, forming the foundation for rapid water uptake. Studies have further clarified that water molecules are initially bound via hydrogen bonding with hydrophilic groups (e.g., –COOH, –OH) on the polymer chains and are subsequently immobilized within the pores through ionic interactions [5]. More critically, the dosage of cross-linkers such as N,N′-methylenebisacrylamide (MBA) has been identified as a key “valve” for tuning the network density: reducing the cross-linking degree expands the network space and enhances the swelling ratio, but an excessive reduction compromises the gel’s structural stability and reusability [3,4]. This progression marks a shift in research focus from solely pursuing high swelling capacity toward achieving an optimal balance among swelling performance, mechanical strength, and structural stability.

Finally, at the functional integration and application-oriented stage, the high water absorption and retention properties of hydrogels have been ingeniously coupled with practical agricultural needs, enabling their functional evolution from simple “water-retaining materials” to “intelligent water nutrient management platforms.” Their core applications are reflected in two key aspects. First, as soil amendments, they significantly enhance soil water retention capacity, particularly in impoverished sandy soils. For example, a cabbage waste-based hydrogel increased the water retention rate of sandy soil by 78.2% and extended the duration of available moisture by five days [3], while a melon peel-based hydrogel remarkably improved the water retention rate of sandy soil by 271.0% [4]. Second, serving as carriers for controlled-release fertilizers, hydrogels utilize their swelling–deswelling behavior to achieve on-demand, slow release of water and nutrients such as urea and zinc ions, thereby significantly reducing nutrient leaching and environmental pollution [5]. Application strategies have also become more refined. For instance, it has been clearly demonstrated that under drought stress, a 1.5% gel application rate represents the optimal concentration for promoting wheat seedling growth, whereas excessive application can inhibit growth due to competition for oxygen and water [3,4]. This dual functionality underscores the transformation of hydrogels into multifunctional platforms that synergistically regulate soil moisture and nutrient release, with precise dosage being critical to maximizing their efficacy in real-world agricultural scenarios.

Research on the water absorption and retention properties of bio-gels has followed a coherent developmental trajectory, progressing from initial raw material selection, through structural regulation, and finally to functional integration. As summarized in Table 1, the water absorption and retention performance of biopolymer hydrogels varies considerably depending on the polymer source, cross-linking strategy, and structural design. For instance, hydrogels such as the cellulose–humic acid composite exhibit a remarkably high swelling capacity of 1007 g/g in distilled water, whereas others like the unmodified PVP-based hydrogel show a much lower value of 31 g/g. Such disparities highlight the critical influence of chemical composition and modification methods on hydrogel performance. A critical analysis of the data in Table 1 further reveals that high swelling capacity does not always correlate with superior water retention. For example, while the melon peel-based hydrogel demonstrates an exceptional swelling capacity of 765.6 g/g, its practical utility depends on its ability to sustain moisture under realistic soil conditions, a property not fully captured by swelling metrics alone. Similarly, the cellulose-based biodegradable hydrogel improves sandy soil water content by 400% at field capacity, yet its retention under wilting point conditions remains limited (10.1%). These observations underscore the necessity of evaluating hydrogels under application-relevant scenarios rather than relying solely on idealized laboratory measurements.

#### 3.1.3. Research Progress in Biocompatibility and Biodegradability

Biocompatibility and biodegradability are core attributes that underpin the safe and effective application of bio-gels in environmental remediation. These properties allow the materials to be assimilated by the environment in an ecologically benign manner after fulfilling their remediation functions, thereby preventing secondary pollution. Early research efforts were primarily dedicated to verifying the basic safety and degradation potential of bio-gels derived from natural sources. Natural and semi-synthetic polymers such as sodium alginate (SA) and polyvinyl alcohol (PVA) gained early attention due to their wide availability, non-toxicity, and ease of gelation. Their interconnected three-dimensional porous structures offer an ideal colonization space for microorganisms, while their biopolymer-based composition facilitates breakdown by environmental microbes. For example, sodium alginate-based gels have been shown to be non-toxic to microbial communities and can ultimately be degraded into CO_2_ and H_2_O by ubiquitous environmental microorganisms [12]. A consensus emerged during this stage that natural-source bio-gels inherently provide a favorable habitat for microorganisms and yield harmless degradation products, aligning with the fundamental principles of green sustainability [37]. However, single-component gels often exhibit insufficient mechanical strength and undergo excessively rapid or unpredictable degradation in complex environments. These limitations constrain the longevity of their remediation function and impede their practical application.

To overcome these limitations, research has advanced into a phase of performance optimization through composite formation and structural modification. The construction of interpenetrating network architectures by combining different materials has emerged as an effective strategy to balance biocompatibility, mechanical strength, and controlled degradation behavior. For instance, a composite system incorporating polyvinyl alcohol (PVA), sodium alginate (SA), and activated carbon (AC) particles was used to fabricate PVA/SA/AC gel microspheres [37]. These microspheres retained excellent biocompatibility and provided a stable microenvironment for the immobilized degrading bacterium *Pseudomonas* sp. DH-6, significantly enhancing its survival rate under harsh conditions such as extreme pH or high salinity compared to free bacteria. More importantly, the degradation rate of the composite gel could be effectively regulated by adjusting the component ratios. As a result, the gel maintained a polycyclic aromatic hydrocarbon (PAH) removal efficiency exceeding 95% even after 90 days of storage or five reuse cycles. This progress signifies a transition in bio-gel development from merely being “safe and usable” toward becoming “stable and controllable”.

**Figure 5:**
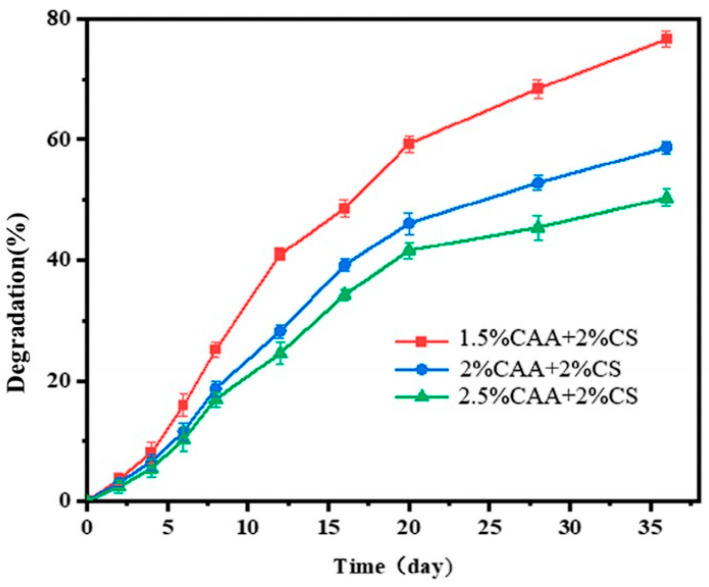
Degradation of Polysaccharide Hydrogels in PBS [38].

Recent advances in green materials science have spurred further research into the biocompatibility and degradability of hydrogels. For instance, Jin et al. [38] demonstrated an inverse relationship between the concentration of cellulose acetoacetate (CAA) and hydrogel degradation rate, where a formulation with 2.5% CAA degraded the slowest, while one with 1.5% CAA degraded the fastest. This behavior is attributed to the denser cross-linked network formed at higher CAA concentrations, which enhances structural stability and decelerates degradation (Figure 5). In the development of sustainable energy storage devices, researchers have systematically evaluated the biocompatibility and controlled degradation behavior of biopolymers including chitosan, cellulose, starch, polylactic acid (PLA), and polycaprolactone (PCL). Standardized soil burial tests and enzymatic degradation studies are widely employed to quantify the environmental degradation rates and ultimate fate of these materials. Studies confirm that systems composed of such materials such as electrodes or electrolytes can safely decompose in natural environments after their service life, significantly alleviating the environmental burden associated with electronic waste [39].

In environmental remediation, biocompatibility extends beyond mere “non-toxicity” to emphasize that the material should not adversely affect soil microbial communities, plant growth, or animal health. Similarly, the concept of degradability is being refined shifting from passive natural degradation toward active and controllable breakdown processes. Inspired by the objectives of green energy devices, future environmental remediation gels should also aim to become intelligent materials with predetermined service lifespans and stimuli-responsive degradation behavior. This would enable on-demand initiation of degradation upon completion of specific remediation tasks, achieving precise full-cycle management from functional deployment to environmentally benign exit.

#### 3.1.4. Advances in Functional Design and Stimuli-Responsive Performance

In early research on heavy metal adsorption using bio-gels, sodium alginate emerged as a key foundational material due to its natural abundance, excellent biocompatibility, and the presence of abundant carboxyl and hydroxyl groups along its molecular chains. Initial studies primarily focused on leveraging its inherent chemical properties to interact with metal ions through mechanisms such as ion exchange and coordination. However, such natural gels often exhibited limitations including low adsorption capacity, poor selectivity, and inadequate mechanical stability.

To address these shortcomings, research has advanced into the phase of surface functionalization and modification. Through methods such as chemical grafting or copolymerization, functional groups with higher specificity such as amino and sulfhydryl groups have been introduced into the sodium alginate network. This strategy significantly enhances both adsorption capacity and selectivity toward specific heavy metals (e.g., Cu^2+^, Pb^2+^, Cd^2+^) [20]. For instance, incorporated amino groups improve copper ion capture via complexation, while sulfhydryl groups form stable covalent bonds with heavy metals such as mercury and cadmium, enabling highly selective adsorption.

Recent advances have focused on integrating biocompatibility with intelligent responsiveness to develop “smart” gels capable of actively adapting to environmental changes. These advanced materials not only inherit the biosafety and biodegradability of natural polymers, but also respond precisely to external stimuli such as temperature, pH, redox conditions, and specific metal ions dynamically modulating their structure and adsorption behavior [40,41].

**Figure 6:**
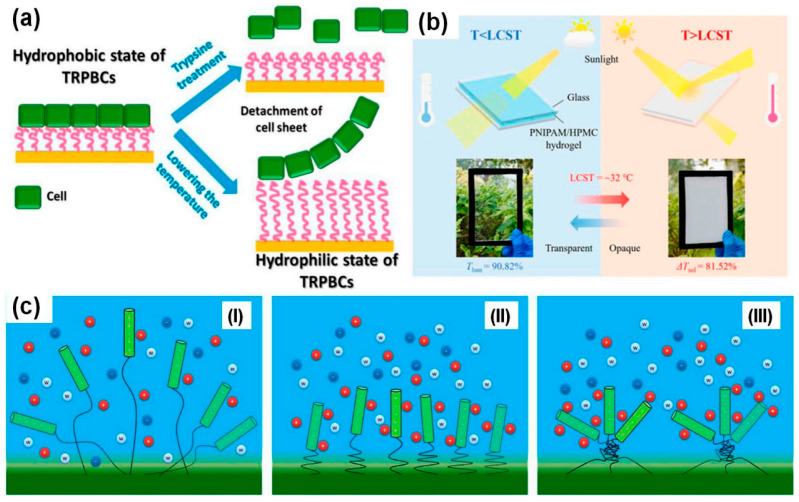
Cell growth (T > LCST) and detachment of the cell sheet from the grafted polymer brushes using trypsin (conventional methodology) or change in temperature (T < LCST) (**a**). Smart window with hydrated PNIPAM film exhibited high light transmittance at T < LCST and bed at T < LCST (**b**). The orientation of DNA molecules (green) conjugated to PNIPAM chains at biosensor surfaces can be controlled with temperature (**c**) with the DNA order parameter: low at T < LCST when hydrophilic polymers extend randomly (**I**); high at the onset of LCST when polymers become hydrophobic and collapse sharply (**II**), and reduced at T > LCST when micro-phase separation of polymers appears (**III**) [43].

For instance, thermosensitive microgels based on poly(N-isopropylacrylamide) (PNIPAM) exhibit reversible hydrophilic/hydrophobic transitions in their molecular chains with temperature variation. Below the lower critical solution temperature (LCST), the gel network swells with open pores, facilitating metal ion diffusion and access to internal functional groups. When the temperature rises above the LCST, the gel rapidly collapses and its pores close. This contraction can trigger the release of adsorbed ions, enabling gel regeneration, while also acting as a reversible “gating” mechanism that controls the adsorption process [42]. Stetsyshyn et al. [43] systematically reviewed the molecular design of dynamic hydrogen bonds and hydrophobic interactions in thermo-responsive polymers, elucidating their central role in the phase transitions at Lower and Upper Critical Solution Temperatures (LCST/UCST) (Figure 6).

Notably, smart gels derived from natural macromolecules offer eco-friendly solutions for environmental remediation. For example, β-cyclodextrin/graphene oxide (β-CD/GO) hybrid gels exhibit both thermosensitive behavior (gel–sol transition between 34.5 °C and 43.5 °C) and selective responsiveness to metal ions such as Na^+^, K^+^, Zn^2+^, Ba^2+^, and Fe^3+^. The disruption of hydrogen bonding between β-CD molecules by these ions induces controlled gel collapse, highlighting the potential of such gels for selective heavy metal adsorption in complex ionic environments [44].

Beyond thermal responsiveness, redox-responsive gels present unique advantages. A representative example is Fc-F hydrogel containing ferrocene groups, which transition from gel to sol upon oxidation by H_2_O_2_, releasing encapsulated pollutants. Subsequent reduction by ascorbic acid restores the gel state, enabling reversible pollutant capture and release [45]. Such systems are particularly suitable for treating wastewater containing oxidizing contaminants.

The integration of functional design with stimulus-responsive mechanisms marks a significant shift from static adsorbents to dynamically controllable platforms for environmental remediation. In particular, multi-responsive gels such as the pH-/thermo-/redox-triresponsive PVA-FPBA-DPH organogel can adapt to complex heavy metal pollution scenarios by responding simultaneously to multiple environmental signals, allowing more precise and efficient remediation [45]. By actively reacting to environmental cues and dynamically modulating adsorption, encapsulation, and release behaviors, these intelligent gel materials offer innovative solutions for efficient and targeted removal of heavy metals under complex environmental conditions [46].

#### 3.1.5. Research Progress in Mechanical Properties and Structural Stability

**Figure 7:**
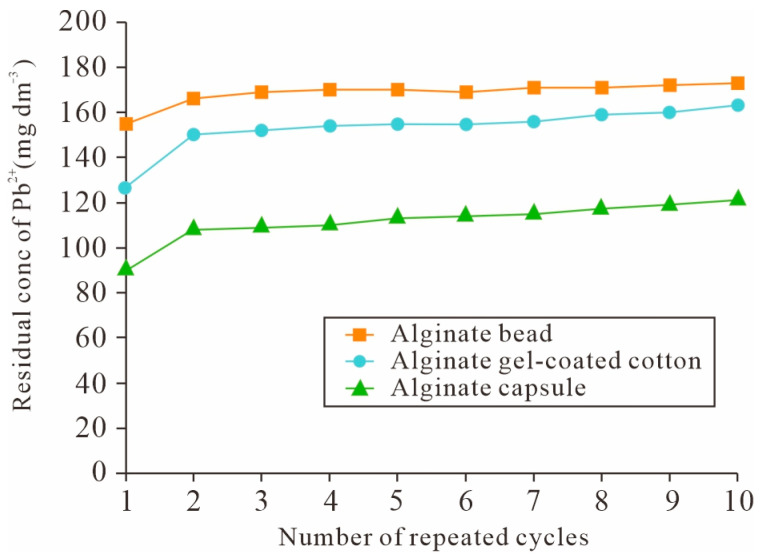
Regeneration of alginate gel-based adsorbents for repetitive Lead (Pb^2+^) adsorption-desorption cycles [12].

The enhancement of mechanical properties and structural stability has been pivotal in advancing bio-gels from laboratory research to practical environmental applications. Early studies predominantly employed chemical cross-linking to construct covalent networks, successfully transforming hydrogels from fluid or soft states into solid materials with defined shapes and moderate mechanical strength. This approach resolved fundamental challenges related to material formation and basic integrity [15]. However, such single-network cross-linked systems often fail to achieve an optimal balance between high adsorption capacity and long-term structural stability. To overcome this limitation, material modification and composite structural design have become key research focuses. For instance, Park et al. [12] developed alginate-based gel adsorbents in the form of microcapsules and gel-coated structures. Their design not only provided excellent metal ion adsorption performance with maximum Pb^2+^ adsorption capacities of 450 mg/g for alginate beads and 1560 mg/g for alginate capsules but also maintained structural integrity with no significant loss in adsorption capacity after 10 adsorption–desorption cycles (Figure 7). This illustrates how rational macrostructural design can synergistically improve both adsorption performance and mechanical durability. Moreover, their study demonstrated that the gel-coated adsorbent achieved adsorption equilibrium within 10 min, significantly enhancing treatment efficiency. The adsorbent could also be stored and applied in a dry state, highlighting its strong potential for real-world water treatment systems.

Furthermore, the incorporation of inorganic nanomaterials has proven to be an effective strategy for enhancing the mechanical properties of bio-gels. For example, composite hydrogels prepared by introducing aminated silica (SiO_2_–NH_2_) into a polyacrylamide (PAM) network exhibit significant improvements in both gel fraction and mechanical strength. Specifically, as the SiO_2_–NH_2_ content increases from 0% to 25%, the gel fraction rises from approximately 91% to nearly 100%, while the mechanical strength is enhanced by severalfold. Notably, the adsorption capacity also increases with higher SiO_2_–NH_2_ content. Under pH = 5 conditions, the Pb(II) adsorption capacity increases from 1.09 mmol/g for pure PAM to 16.2 mmol/g for the composite containing 25% SiO_2_–NH_2_. The experimental values substantially exceed theoretical predictions, indicating a pronounced synergistic coordination effect. Moreover, the composite hydrogel demonstrates excellent stability over multiple regeneration cycles, with a desorption ratio exceeding 97% and no significant decline in adsorption capacity for Pb(II), Cu(II), and Cd(II) after repeated adsorption desorption cycles [25]. This organic inorganic composite strategy not only reinforces the gel mechanically through physical incorporation but also enhances heavy metal adsorption via synergistic coordination provided by functional groups on the nanoparticle surfaces.

Although strategies such as composite design and nanomaterial reinforcement have significantly enhanced the mechanical properties of bio-gels, their structural stability remains a considerable challenge in practical applications particularly under complex environmental conditions. Field trials and large-scale implementations have shown that bio-gel structures can be compromised by physical stress, chemical erosion, or biodegradation, leading to functional failure and potential secondary pollution. A representative case is the attempt to encapsulate photocatalytic nanoparticles within gel matrices to create dual-functional materials capable of both adsorption and degradation. As demonstrated by Mazzeo et al. [47], calcium alginate gel beads loaded with a high concentration (10% *w*/*w*) of TiO_2_ nanoparticles suffered severe structural instability under UV irradiation. Although the TiO_2_ nanoparticles exhibited high photocatalytic activity in degrading methylene blue, the free radicals generated during the process simultaneously attacked and degraded the alginate network. Spectrophotometric analysis confirmed matrix breakdown, manifested as the gradual release of nanoparticles, which altered the background absorbance of the solution. This case clearly illustrates that when functional units (e.g., nanoparticles) within the gel are excessively active or highly concentrated, their reactive byproducts such as free radicals can damage the gel matrix, resulting in premature structural collapse. Such issues not only limit the reusability of these composite materials in photocatalytic applications but also underscore the importance of considering chemical compatibility and long-term stability between the gel matrix and functional components during material design.

Meanwhile, the strategic selection of cross-linker types shows great potential for precisely tuning gel stability. Mishra et al. [15] synthesized polyacrylic acid-based hydrogels using novel bio-based cross-linkers such as allyl sorbitol and allyl mannitol. These hydrogels not only maintain high adsorption capacities but also exhibit excellent structural integrity and reusability. Specifically, they achieved adsorption capacities of 1.69 mmol/g for Pb^2+^, 0.69 mmol/g for Cu^2+^, and 0.51 mmol/g for Hg^2+^. After ten adsorption–desorption cycles, the hydrogels retained desorption efficiencies of approximately 88% for Pb^2+^, 82% for Cu^2+^, and 72% for Hg^2+^, demonstrating robust regeneration performance. These findings indicate that rational design of cross-linker structures can significantly enhance the environmental adaptability and service life of gels without compromising their adsorption function.

Therefore, future research should place greater emphasis on the performance of bio-gels under real-world conditions, systematically evaluating their resistance to mechanical wear, chemical oxidation, microbial degradation, and internal active component erosion throughout their operational lifespan. Such comprehensive assessment is essential to advance the translation of bio-gels from laboratory research to practical engineering applications.

### 3.2. Functions of Bio-Gels

#### 3.2.1. Heavy Metal Ion Adsorption

Bio-gels interact with heavy metal ions through multiple functional groups such as carboxyl, amino, and hydroxyl groups within their three-dimensional network structures. Bio-gels employed for metal ion remediation generally exhibit high adsorption capacity, broad raw material availability, and low cost [48]. The primary adsorption mechanisms include:

(1) Ion Exchange: Ions present in the gel network are displaced by heavy metal ions such as Pb^2+^, Cd^2+^, and Cu^2+^ from the surrounding solution [49,50]. (2) Coordination: Functional groups including amino and carboxyl groups donate lone pair electrons to form stable coordination bonds with vacant orbitals of heavy metal ions. This represents the core adsorption mechanism in gels such as chitosan [49]. (3) Electrostatic Adsorption: Under specific pH conditions, the charged gel surface attracts metal complex ions of opposite charge through electrostatic interactions [49].

**Figure 8:**
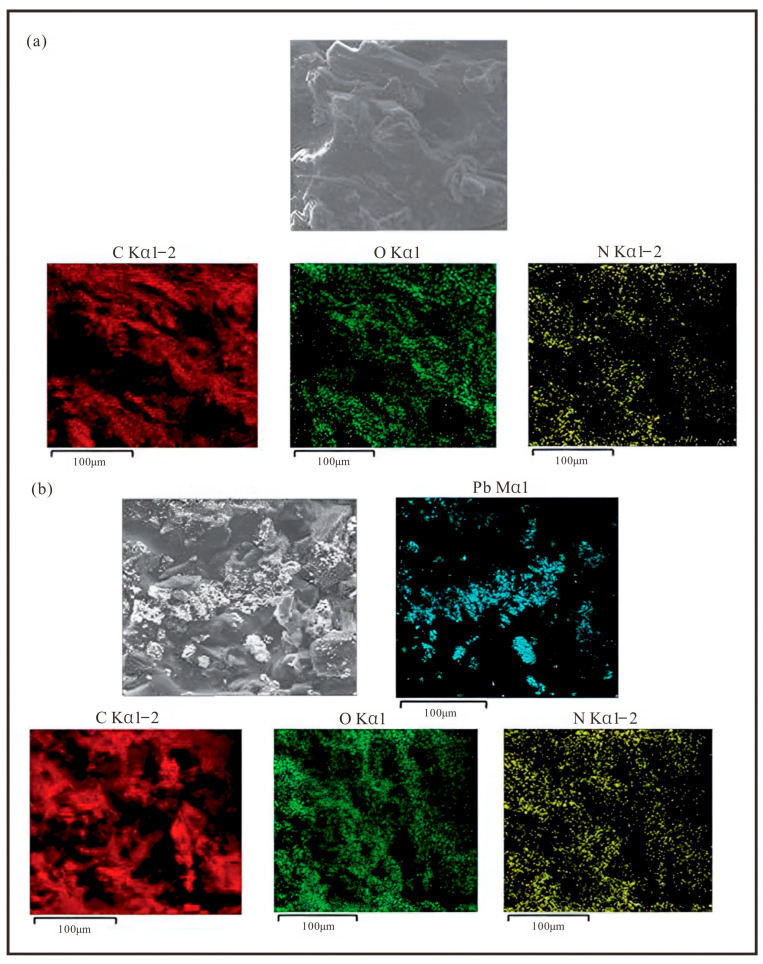
EDX dot mapping of GE/AC (**a**) before and (**b**) after adsorption of Pb^2+^ ions. EDX: energy dispersive X-ray spectroscopy; GE/AC: gelatin/activated carbon [51]. The blue bright points represented the Pb2þ ions adsorbed on GE/AC.

Several studies have summarized the adsorption performance of various bio-gels for heavy metal ions, as summarized in Table 2. For instance, polyvinyl alcohol (PVA)/gelatin hydrogel beads have been applied for Pb^2+^ removal. It was observed that a higher gelatin content resulted in a looser network structure, which improved material permeability and enhanced Pb^2+^ adsorption capacity [51,52], as illustrated in Figure 8. Table 2 consolidates the adsorption performance of various bio-gels for heavy metal ions, revealing significant disparities in capacity that warrant critical analysis. The maximum adsorption capacities span nearly two orders of magnitude, from ~68.52 mg/g for Cd^2+^ on cross-linked chitosan gel beads to an exceptional 1560 mg/g for Pb^2+^ on alginate gel capsules. This vast range underscores the profound influence of gel composition and structure. For instance, the incorporation of high-capacity polymers like polyethyleneimine (PEI) in ternary composite beads or the creation of macroporous, gel-coated architectures can drastically enhance performance compared to single-component networks. However, these direct comparisons are often complicated by inconsistent experimental parameters across studies. Critical factors such as initial concentration, adsorbent dosage, and solution pH vary considerably, making it difficult to establish a unified performance benchmark. Furthermore, while many gels exhibit a preferential adsorption order of Pb^2+^ > Cu^2+^ > Cd^2+^, attributed to the higher electronegativity and hydration energy of Pb^2+^, their selectivity in multi-metal systems is often inadequately reported. The long-term practical utility of these materials is another critical consideration. Although several entries, such as the PAM/SiO_2_–NH_2_ composite, report excellent reusability, the gradual decline in capacity over multiple cycles—A common challenge—is not always quantified. Therefore, a critical assessment of Table 2 highlights that future research must not only pursue higher adsorption capacities but also systematically report and optimize performance under standardized conditions, with a focus on selectivity, kinetics, and long-term stability for practical application.

#### 3.2.2. Microbial Immobilization

Bio-gels serve not only as a protective microenvironment for functional microorganisms such as degrading bacteria and nitrifying/denitrifying bacteria by shielding them from harsh conditions including toxic compounds and pH fluctuations [13,15], but also enhance their metabolic activity and operational stability [56]. Moreover, they enable the targeted localization and retention of high concentrations of functional microorganisms in contaminated zones [57]. Early microbial immobilization studies often relied on single-component materials (e.g., calcium alginate) for simple physical entrapment. While straightforward to prepare, these systems generally exhibited poor mechanical strength, susceptibility to degradation, and limited functionality. However, recent advances in material design have substantially improved immobilization performance through the development of composite and functionally tailored carrier systems [37].

To further extend the functionality of immobilized systems and address complex contamination scenarios, researchers have begun developing intelligent gel carriers with active environmental responsiveness and enhanced capabilities. Zhang et al. [18] immobilized nitrate-reducing Fe(II)-oxidizing bacteria in calcium alginate hydrogels and observed that the three-dimensional network structure effectively alleviated the encapsulation stress and mineral-induced toxicity to cells during Fe(II) oxidation. As a result, the nitrate reduction rate in the immobilized system was approximately 3.8 times higher than that of free bacterial systems. The study further revealed that the alginate skeleton itself can serve as an additional carbon source for microorganisms, promoting Fe(II)-independent nitrate reduction. This innovative “carrier-as-nutrient” strategy offers a promising solution for treating wastewater with low carbon-to-nitrogen ratios, achieving over 95% nitrate removal from simulated wastewater in their experiments.

In summary, microbial immobilization technology has evolved from simple early-stage encapsulation to contemporary systems characterized by composite materials, multifunctional integration, and stimuli-responsive design. Through deliberate material design, researchers have not only enhanced the physical protection of microorganisms but also endowed these systems with improved mass transfer efficiency, environmental tolerance, reusability, and even additional remediation functions such as adsorption and nutrient supply. These advances have significantly expanded the applicability and effectiveness of bioaugmentation in complex environmental remediation scenarios.

#### 3.2.3. Soil Improvement and Ecological Restoration

**Figure 9:**
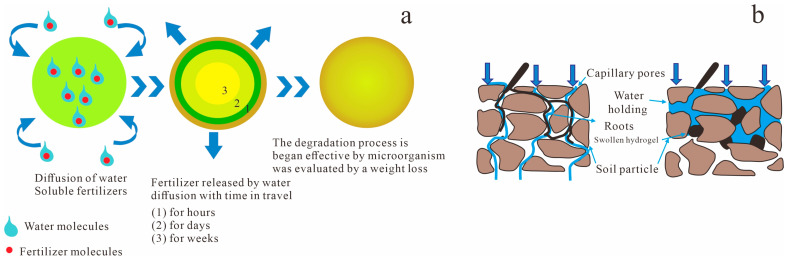
(**a**) An illustration of the complete fertilizer release procedure from hydrogel (**b**) the impact of polysaccharide hydrogel on the soil texture for plant growth [3].

As an efficient soil conditioner, bio-gels show considerable potential in environmental remediation. Their effectiveness stems primarily from the remarkable water absorption and retention capacities enabled by their three-dimensional network structure. This allows gradual water release under arid conditions, significantly reducing soil evaporation losses, improving water-use efficiency, and simultaneously enhancing soil aggregation, increasing porosity, and promoting root development [3,4]. Bio-based hydrogels derived from natural polymers such as starch, chitosan, sodium alginate, and lignin have enabled the development of novel gel systems that combine high water absorption with environmental compatibility. For instance, starch-grafted acrylic acid hydrogels not only improve soil moisture retention but also serve as controlled-release carriers for fertilizers, facilitating integrated water and nutrient management [3] (Figure 9).

Moreover, the resource utilization of agricultural waste has opened a new pathway for the green preparation of bio-gels. Studies have shown that hydrogels derived from fruit residues such as melon peel and orange peel not only exhibit high water absorption capacity (up to 765.6 g/g) but also significantly enhance the water retention of sandy soil, promoting normal wheat growth under drought stress [4]. This “turning waste into anti-desertification” strategy simultaneously addresses agricultural waste disposal while offering a low-cost and sustainable water-management solution for crop cultivation in arid regions.

Under extreme soil conditions, bio-gels demonstrate enhanced functionality in ecological restoration [58]. For instance, a cellulose-based “gel–glycerol” hydrogel maintained an ultra-high water retention rate (160%) even in saline–alkali and sandy soils, significantly improving the germination rate and biomass of wheat and lettuce. This effect is attributed to the gel network’s ability to regulate the distribution of trace elements such as Zn^2+^ in the soil, thereby improving the rhizosphere microenvironment [59]. Similarly, in arid and semi-arid ecological restoration projects, polyaspartic acid (PASP) hydrogel increased seedling survival rates by 8–12% and elevated leaf relative water content by 4–16%. It also alleviated drought stress-induced inhibition of the photosynthetic system by improving chlorophyll fluorescence parameters [60].

#### 3.2.4. Controlled Release of Active Agents

Leveraging the environmental responsiveness of bio-gels such as to pH, enzymes, temperature, and moisture researchers have developed functional systems that encapsulate pesticide or fertilizer molecules within their three-dimensional networks. This enables the slow and controlled release of active ingredients through diffusion, swelling, erosion, or degradation, representing a significant strategy in environmental remediation and sustainable agriculture [61]. Early research primarily focused on simple physical encapsulation. For example, Muharam et al. [62] developed a superabsorbent hydrogel by cross-linking corncob cellulose with epichlorohydrin and used it to store urea within its porous structure. This method achieved a preliminary delay in urea release, with only 5.29–5.56% released within 180 min, highlighting the potential of bio-gels as carriers to improve fertilization efficiency and reduce environmental impact.

As research has advanced, the focus has shifted from “passive encapsulation” to “active regulation.” By precisely designing hydrogel composition and structural parameters, it is now possible to systematically control gel strength and release behavior. Wei et al. [63] established for the first time a quantitative relationship between gel strength and urea release kinetics in starch-based hydrogels. They found that reducing urea loading, increasing the proportion of grafted acrylamide, or raising the cross-linker (MBA) concentration significantly enhanced gel strength, thereby effectively suppressing the initial burst release. This adjustment reduced the first-day cumulative release rate from 78% to 52%. Their study not only revealed the influence of gel network heterogeneity on release behavior but also proposed a “gel strength release behavior” regulation strategy, providing theoretical and experimental foundations for the rational design of high-performance slow-release materials.

Chitosan-based gels are regarded as ideal carriers for intelligent controlled-release systems due to their excellent biocompatibility, modifiability, and multi-responsive characteristics. Liu et al. [61] systematically reviewed various preparation and loading strategies for chitosan gels in nutrient delivery, including adsorption, homogeneous dispersion, core shell encapsulation, and intercalation composites. They emphasized that release behavior can be described by multiple models such as Fickian diffusion, swelling control, and network erosion. For instance, introducing pH-sensitive groups (e.g., carboxymethyl) or thermo-responsive units (e.g., poly(N-isopropylacrylamide)) enables on-demand nutrient release in response to soil microenvironmental changes, significantly improving spatiotemporal release precision.

In summary, bio-gels exhibit multifaceted functional characteristics in environmental remediation: As heavy metal adsorbents, their three-dimensional networks rich in functional groups (e.g., carboxyl and amino) enable efficient ion removal via ion exchange, coordination, and electrostatic adsorption, offering high capacity and regenerability. As microbial immobilization carriers, they provide physical protection for functional microorganisms while enhancing metabolic activity and degradation efficiency through intelligent design for example, alginate gels serving simultaneously as a carbon source increased the nitrate reduction rate by approximately 3.8-fold. As soil conditioners, their superabsorbent and water-retaining properties improve soil structure and moisture retention. Agricultural waste-derived gels have been shown to promote crop growth in sandy soils, exemplifying a “treating waste with waste” strategy. As controlled-release carriers, their environmental responsiveness enables slow and targeted release of pesticides or fertilizers. By tuning gel strength and network structure, nutrient loss is reduced and utilization efficiency improved. Collectively, these functionalities underscore the integrated application value of bio-gels in pollution control and ecological restoration (Table 3).

## 4. Directions for Expanding the Application Fields of Bio-Gels

Owing to their unique physicochemical properties such as high water content, three-dimensional porous network structure, excellent biocompatibility, and tunability bio-gels have rapidly expanded their applications from the traditional medical field to emerging frontier areas, including environmental engineering and energy development.

### 4.1. Remediation in Industrial Applications

#### 4.1.1. Heavy Metal Adsorption

Natural bio-gels such as sodium alginate and chitosan, which are rich in functional groups (e.g., carboxyl and amino groups), have shown considerable effectiveness in adsorbing heavy metal ions (e.g., Cu^2+^, Cd^2+^, Pb^2+^, Cr^6+^) from industrial wastewater via mechanisms including ion exchange and complexation [24]. Recent advances in functional modification have further enhanced their adsorption performance, as evidenced by several key studies that highlight both their capabilities and limitations.

For instance, Cao et al. [21] developed hydrolyzed polyacrylamide–chitosan composite gel beads with a Pb^2+^ adsorption capacity of 1.69 mmol/g. Although competitive among biopolymer-based adsorbents, this value remains lower than some fully synthetic or hybrid systems, highlighting a typical trade-off between biodegradability and maximum uptake. Moreover, while the material retained 88% desorption efficiency after ten cycles demonstrating robust reusability the gradual decline in performance suggests progressive loss of active sites due to functional group degradation or pore blockage, which requires further investigation for long-term use.

In a separate study, Wang et al. [25] synthesized polyacrylamide/modified silica composite hydrogels that exhibited a synergistic adsorption effect, with experimental uptake exceeding theoretical predictions. This enhancement is attributed to cooperative interactions between functional groups on the polymer and modified silica, leading to more efficient metal complexation. Notably, adsorption equilibrium was achieved within 10 min, indicating rapid kinetics suitable for continuous-flow systems. However, the high silica content, while improving mechanical strength, may reduce swelling capacity and limit accessibility to internal adsorption sites a potential limitation in diffusion-dominated contexts. Regarding regeneration, the same composite hydrogel showed a desorption rate exceeding 97% under mild acid treatment and maintained high adsorption capacity over multiple cycles. Nevertheless, the gradual performance decline consistent with the observations of Cao et al. [21] points to a common challenge in hydrogel adsorbents: the progressive deterioration of active sites during repeated use. This underscores the need for future research to enhance the structural and chemical resilience of bio-gels under cyclic operating conditions.

In summary, while modified bio-gels exhibit clear advantages in adsorption capacity, kinetics, and reusability, a critical assessment reveals persistent limitations. These include capacity ceilings relative to non-biological adsorbents, inherent trade-offs between component ratios and overall performance, and gradual efficiency loss during cyclic operation. Addressing these challenges will be essential to advancing the practical application of bio-gels in industrial wastewater treatment.

#### 4.1.2. Adsorption of Organic Dyes

Natural polymer-based hydrogels have demonstrated significant potential for removing organic dyes from wastewater, though their performance is strongly influenced by structural design and the accessibility of functional groups. A critical analysis of recent studies illustrates how compositional and architectural variations govern adsorption efficiency and practical applicability.

Kim et al. [8] developed a metal-ion-free κ-carrageenan/cellulose hydrogel bead system using an ionic liquid mixture. By avoiding conventional metal-ion cross-linking, the anionic sulfate groups of κ-carrageenan were preserved, enhancing electrostatic interaction with cationic dyes. The beads showed a high initial swelling ratio of 28.3 g/g and retained over 50% of their adsorption capacity after drying a practical advantage for storage and reuse. The maximum adsorption capacity for crystal violet reached 241 mg/g, with a clear linear correlation between κ-carrageenan content and adsorption performance, confirming the predominance of electrostatic mechanisms. This system outperforms many reported κ-carrageenan-based adsorbents, such as those cross-linked with K^+^, which often exhibit reduced sulfate group availability. However, the notable decline in swelling and adsorption capacity after drying indicates structural irreversibility, which may constrain practical utility in repeated cycles unless intermediate rehydration is applied.

By contrast, Guo et al. [6] developed a supramolecular gel based on glycyrrhetinic acid and Cu^2+^, which exhibited Cu^2+^-triggered shrinkage, enabling self-demolding and improved mechanical integrity. The gel achieved an equilibrium adsorption capacity of 82.92 mg/g for methylene blue, primarily through electrostatic interactions, as evidenced by zeta potential and FT-IR analyses. Although its adsorption capacity is lower than that of the κ-carrageenan/cellulose system, the gel displayed strong antimicrobial activity against Gram-positive bacteria including MRSA underscoring its dual functionality in combined wastewater treatment. Reusability remained excellent over four cycles with minimal performance loss. However, the dependence on Cu^2+^ may pose environmental concerns, and its selectivity for cationic dyes, while advantageous in specific contexts, limits its applicability in mixed-dye effluents.

When critically evaluated, the κ-carrageenan/cellulose system offers superior adsorption capacity and avoids metal ions, making it more environmentally compatible and suitable for high-load scenarios. Its main drawback lies in structural collapse upon drying, requiring careful handling. The glycyrrhetinic acid-based gel, though less adsorptive, provides supplementary antibacterial function and enhanced mechanical stability through metal coordination representing a trade-off between capacity and multifunctionality. Both systems follow pseudo-second-order kinetics and Langmuir-type adsorption, indicating chemisorption-dominated mechanisms. Their practical deployment will ultimately depend on the target dye type, water matrix composition, and sustainability priorities. Future research should aim to reconcile the stability–capacity trade-off and explore composite designs that integrate high adsorption performance with secondary functionalities such as antimicrobial or photocatalytic properties.

### 4.2. Remediation in Agricultural Applications

#### 4.2.1. Water Retention/Holding Capacity

Water retention capacity serves as a key performance indicator for evaluating bio-hydrogels in agricultural applications. Current research demonstrates that bio-hydrogels not only significantly improve soil water retention but also show potential for functional expansion. However, their effectiveness is closely tied to material composition, structural design, and soil type, necessitating context-specific evaluation. For example, the hydrogel developed by Xie et al. [5] from Chinese cabbage waste increased the water retention capacity of sandy soil by 78.2% and extended the duration of available moisture by five days, highlighting its suitability for arid-region agriculture. In another study, Fang et al. [4] reported that a melon peel-based hydrogel improved the water retention rate in sandy soil by 271.0%, substantially outperforming most conventional water-retaining materials as a result, attributed to its high cellulose content and optimized cross-linked structure. It is noteworthy that hydrogels derived from different feedstocks exhibit varying performance across soil types, emphasizing the need for tailored selection based on specific application contexts.

Beyond water retention, bio-hydrogels have also progressed in multifunctional integration. Zheng et al. [7] developed an alginate/lignin composite gel with limited swelling, which acts as both a “smart pesticide carrier” and a “heavy metal scavenger”. This system enables targeted pesticide release under reducing conditions while effectively adsorbing Pb^2+^ ions, offering synergistic benefits for the remediation of contaminated soils. Nevertheless, the practical performance of such systems under real field conditions requires further validation, particularly regarding their stability and controllability in complex soil microbial environments. In summary, while bio-hydrogels demonstrate clear advantages in enhancing soil water retention and integrating multiple functions, future studies should place greater emphasis on long-term performance evaluation and economic feasibility in real agricultural settings to facilitate their translation from laboratory research to field application.

#### 4.2.2. Ecological Environment Restoration

Beyond their role in water management, bio-gels serve as efficient and precise delivery systems for agrochemicals. Ungureanu et al. [64] highlighted several successful applications of cellulose-based hydrogels in the controlled release of fertilizers and pesticides. For instance, a hydrogel synthesized from cotton straw composed of cellulose-g-poly(acrylic acid) and bentonite exhibited a nitrogen leaching loss of only 4.8%, significantly lower than the 43% loss observed with conventional urea application. In pesticide delivery, loading the herbicide acetochlor into composite gels of carboxymethyl cellulose (CMC) and various clays extended the time required for 50% release of the active ingredient to 151–522 h, compared to the rapid release of non-encapsulated herbicide. This sustained-release behavior not only improves the utilization efficiency of fertilizers and pesticides and mitigates non-point source pollution from runoff and leaching but also supports in situ immobilization and remediation by reducing the concentration of freely available pollutants in soil.

The application of bio-gels is evolving from single-component materials toward integrated and intelligent systems. Kabir et al. [65] developed a real-time soil moisture monitoring system that combined a DMAA hydrogel with low-cost sensors and an Arduino microcontroller. This system successfully tracked sustained higher soil moisture levels over multiple days in gel-amended soil, offering both data support and a technical prototype for precision irrigation on demand. In a broader closed-loop approach, Zhou et al. [66] created an “atmospheric water irrigation system”. This system captures and stores atmospheric moisture at night using a SMAG–soil composite and releases it during the day via solar energy. It maintained a relative humidity of approximately 70% in the planting chamber, even when external humidity dropped as low as 30%. When radish was cultivated in this system using only atmospheric water, plants reached about 15 cm in height with 100% survival after 14 days. By contrast, radish grown in unamended sandy soil reached only about 1 cm and did not survive beyond 6 days. These results clearly illustrate the potential of intelligent bio-gel systems to significantly enhance crop survival and growth under extreme environmental conditions.

### 4.3. Applications in Oilfield Development

In the field of oilfield development, bio-gels are emerging as sustainable alternatives to conventional chemical agents due to their biodegradability, environmental compatibility, and stimuli-responsive behavior. These materials can respond precisely to downhole conditions such as pH, temperature, and mechanical stress enabling intelligent control over functions including fluid loss prevention, enhanced oil recovery, and corrosion inhibition [67]. Compared to traditional chemical agents, bio-gels not only offer more precise and controllable performance but also reduce long-term risks of formation damage, aligning with the green development objectives of the oil and gas industry.

Depending on their response mechanisms, different types of smart bio-gels exhibit distinct application advantages: pH-responsive gels, such as those based on AM/AMPS copolymers, can expand up to 17.8 times in alkaline environments, enabling delayed swelling and effective sealing. Stress-responsive gels, exemplified by PVA/chitosan dual-network systems, combine high mechanical strength with self-healing capability, making them suitable for long-term plugging in high-temperature and high-pressure well conditions [68]. Temperature-responsive gels operate through thermally triggered release mechanisms. For instance, gelatin-based biocomposite gels allow on-demand release of corrosion inhibitors, providing adaptive protection for downhole pipelines [67]. In enhanced oil recovery, alkaline-responsive gel microspheres based on cellulose nanocrystals (CNC) can selectively plug high-permeability zones and adjust mobility ratios, effectively displacing residual oil and significantly improving crude oil recovery [69,70].

The successful development of these stimuli-responsive bio-gels marks a strategic shift in oilfield chemical agents toward precision and intelligence. As the understanding of subsurface response mechanisms deepens and material innovations continue, bio-gels are expected to play increasingly critical roles in more complex oilfield development scenarios. Future research will focus on designing gel systems capable of synergistic responses to multiple environmental signals, enhancing their long-term stability under extreme downhole conditions, and establishing comprehensive life-cycle environmental safety assessment frameworks. These advances will provide robust technical support for the sustainable development of the oil and gas industry.

### 4.4. Applications in Biomedical Engineering

In the field of biomedical engineering, bio-gels particularly hydrogels characterized by high water content and three-dimensional network structures have become essential functional materials. Their key advantage lies in their ability to closely mimic the physicochemical properties of the natural extracellular matrix, providing cells with a hydrated, soft, and mechanically compatible microenvironment. At the same time, their porous architecture permits the free transport of nutrients, metabolic waste, and signaling molecules. These characteristics enable hydrogels to serve critical functions in tissue engineering, drug delivery, and wound healing [71].

Chitosan-based bio-gels have gained considerable interest as advanced wound dressings owing to their inherent biocompatibility, biodegradability, and antimicrobial activity. In addition to creating a moist wound microenvironment that supports healing, these gels serve as sophisticated delivery platforms that can encapsulate and controllably release drugs, growth factors, or therapeutic cells often in response to wound-specific stimuli such as pH changes or enzymatic activity [19]. For example, recent studies have shown that chitosan hydrogels not only promote the survival and proliferation of bone marrow mesenchymal stem cells (BMSCs) but also enhance their paracrine signaling, resulting in accelerated vascularization and tissue regeneration in burn models [72]. In comparative animal studies, chitosan-BMSC hydrogel composites demonstrated a wound closure rate over 40% faster, along with significantly higher capillary density, compared to conventional gauze or chitosan-only treatments. These findings highlight their functional superiority in promoting regenerative outcomes. Despite these promising results, several challenges remain inadequately addressed in the current literature. Variations in chitosan source, deacetylation degree, and molecular weight hinder direct comparison of hydrogel performance across studies. Furthermore, most reported systems lack long-term stability data under physiological conditions, and their mechanical strength often proves insufficient for dynamic wound environments.

Furthermore, the emergence of stimuli-responsive smart hydrogels has significantly advanced the precision of drug delivery systems. These intelligent materials can detect specific changes in pathological microenvironments such as acidic pH in tumor regions, elevated enzyme levels due to inflammation or infection, and abnormal redox states in diseased tissues and respond through swelling, shrinking, degradation, or structural transformation. This enables on-demand, targeted, and controlled drug release [73]. For instance, pH-sensitive hydrogels can maintain structural stability in the acidic gastric environment while releasing encapsulated drugs in the neutral pH of the intestines. Enzyme-responsive hydrogels, by contrast, undergo specific cleavage in the presence of elevated matrix metalloproteinase concentrations around tumor sites, allowing localized delivery of anticancer agents.

Recent advances in modular protein design have enabled the development of smart hydrogels with programmable stimulus-responsive behavior. Grove et al. [74] constructed a hydrogel system based on tetratricopeptide repeat (TPR) protein arrays cross-linked with multi-arm PEG–peptide conjugates. Gelation and dissolution are governed by electrostatic interactions between TPR modules and their cognate peptide ligands, conferring ion-strength-dependent responsiveness. These gels remain stable under low-salt conditions, exhibiting an elastic modulus of up to 270 Pa sufficient to support suspended cells while undergoing controlled degradation in high-salt environments. The system efficiently encapsulates both a 26 kDa fluorescent protein and small drug-mimetic molecules such as rhodamine, and enables tunable release kinetics under physiologically relevant ion strengths: while macromolecular release coincides with gel erosion, small molecules are released more rapidly. This behavior highlights the potential of such systems for synergistic delivery of protein-based and small-molecule therapeutics. The spatiotemporally controlled release profiles not only improve the therapeutic index but also reduce off-target effects, underscoring the value of these materials as a foundational platform for precision medicine.

The application of bio-gels also demonstrates significant potential in the regeneration and repair of hard tissues. For instance, Li et al. [75] developed a bio-gel enriched with autologous regeneration factors (ARF) to treat osteonecrosis of the femoral head (ONFH). The ARF bio-gel effectively mimics the natural extracellular matrix, providing a favorable microenvironment that supports the proliferation and osteogenic differentiation of bone marrow mesenchymal stem cells (BMSCs). Furthermore, in a large animal (goat) model, the gel significantly promoted bone regeneration and functional recovery by modulating macrophage polarization toward the M2 phenotype, regulating the inflammatory response, and stimulating vascular remodeling within necrotic areas. This study highlights the capacity of bio-gels to serve as multifunctional scaffolds that enable in situ tissue regeneration under complex pathological conditions, substantially broadening their applicability in regenerative medicine.

## 5. Future Prospects

Despite considerable advances in bio-gel research, further in-depth exploration is required in the following key directions to enable large-scale practical application and industrialization:

(1) Elucidating microscopic interaction mechanisms

Advanced techniques such as synchrotron radiation, high-resolution microscopy (e.g., cryo-SEM), spectroscopy, and molecular dynamics simulations should be employed to investigate the transport, adsorption, and degradation kinetics of pollutants (especially complex contaminants) within gel networks at molecular and nanoscales. Understanding their precise interactions with functional groups is essential for the rational design of novel gel materials with high selectivity and capacity.

(2) Developing low-cost and green preparation processes

Greater emphasis should be placed on using renewable resources such as agricultural waste and industrial byproducts as raw materials. Green synthesis pathways including aqueous synthesis and enzyme-catalyzed cross-linking should be adopted to achieve low-carbon and economical material production. Promoting a “waste-treats-waste” circular economy model and establishing a complete technological chain from raw material selection and process optimization to scaled-up production will significantly enhance the market competitiveness of bio-gels.

(3) Precise design of multifunctional stimuli-responsive gels

To meet complex environmental remediation needs, intelligent gels capable of responding simultaneously to multiple signals such as pH, temperature, ionic strength, specific pollutants, and enzymes should be developed. Through molecular-level design, materials can achieve synergistic responses and adaptive regulation to external stimuli, enabling “smart” remediation systems with feedback functions. This will shift environmental remediation from passive treatment toward precision control.

(4) Enhancement of structural stability and long-term service performance

To overcome limitations such as insufficient mechanical strength, structural collapse in complex environments, and rapid degradation, strategies including dual/multi-network structures, incorporation of nanocomposites (e.g., cellulose nanocrystals, modified silica), and optimization of cross-linking topology should be pursued. These approaches can significantly improve mechanical robustness, swelling stability, and fatigue resistance, thereby extending service life and reusability in field applications.

(5) Systematic assessment of environmental safety and full life-cycle ecological risks

Prior to widespread deployment, it is essential to systematically evaluate the transport, transformation, and accumulation of bio-gels and their degradation intermediates in environmental media (water, soil, sediment), as well as their long-term effects on plants, animals, microbial communities, and ecosystem functions. Establishing scientifically sound and comprehensive environmental safety evaluation standards and frameworks is crucial for ensuring the sustainable development of bio-gel technologies.

Through sustained innovation and breakthroughs in these areas, bio-gels are expected to play an increasingly vital role in future environmental governance systems, providing robust technical support for addressing global challenges of pollution and ecological degradation.

## 6. Conclusions

Biopolymer gels, with their tunable three-dimensional network structures, have emerged as versatile and sustainable materials for environmental remediation. Their effectiveness stems from a well-defined “structure-property-function” relationship: an interconnected porous architecture offers a high specific surface area and facilitates pollutant transport and microbial colonization, while abundant functional groups such as carboxyl, amino, and hydroxyl enable efficient contaminant removal via ion exchange, coordination, and electrostatic interactions. In addition, their inherent biocompatibility and adjustable biodegradability support environmentally safe application. A multi-dimensional classification framework based on origin, cross-linking mechanism, and functional traits enables the rational design of gels tailored to specific remediation needs. This has led to expanding use across industrial, agricultural, oilfield, and biomedical sectors.

Despite these advances, challenges persist in improving mechanical stability, scaling up production, and ensuring long-term environmental safety. Future efforts should prioritize elucidating molecular-level interaction mechanisms, developing low-cost and green synthesis routes, designing multi-stimuli-responsive intelligent gels, and establishing comprehensive life-cycle assessment protocols. Addressing these issues will solidify the role of biopolymer gels as efficient and sustainable solutions to complex environmental problems.

## Figures and Tables

**Table 1:** Water Absorption and Retention Data of Biopolymer Hydrogels.

No.	Hydrogel Type	Water Absorption Performance Data	Water Retention Performance Data	References
1	Cellulose-based biodegradable hydrogel	Sandy soil with 2% hydrogel: water content at field capacity (pF 2.5) increased by 400%	At wilting point (−15 bar), soil with 2% hydrogel retained 10.1% water, equivalent to field capacity of unamended soil	[29]
2	Cellulose–humic acid composite hydrogel	Swelling capacity: 1007 g/g in distilled water; 143 g/g in 0.9% NaCl solution	Water retention rate: 79.2% after 24 h; 22.5% after 72 h at room temperature	[30]
3	Gleditsia microphylla galactomannan hydrogel	Swelling index: 88.70 g/g at pH 9; 54.00 g/g in 0.9% NaCl; 77.72 g/g in tap water	Water-holding time in sandy soil extended from 3 to 23.5 days (0.5% hydrogel); stable over 10 absorption–retention cycles	[31]
4	PVP-based multifunctional hydrogel	Swelling degree: untreated hydrogel: 31 g/g; NaOH-treated: 207 g/g; KOH-treated: 315 g/g	Initial water retention ~40%; hydrogel #2 released water within 3–4 days; sustained release of N, P, K over 10 days	[32]
5	Melon Peel-based Hydrogel	Swelling capacity: 765.6 g/g (in ultrapure water)	Increased sandy soil water-holding capacity by 271.0%	[4]
6	Chinese Cabbage Waste-based Hydrogel	Swelling capacity: 551.8 g/g (in ultrapure water)	Increased sandy soil water-holding capacity by 78.2%; extended water retention time by 5 days.	[5]
7	Banana Peel-based Hydrogel	Swelling capacity:495.0 g/g (in ultrapure water)	Increased clay soil water-holding capacity by 43.3%.	[5]
8	LBG/Borax Hydrogel	Maximum Water Absorption Capacity in deionized water: 130.29 g/g	Maximum Soil Water Content increased by: 32.03%; Soil Water Retention Time extended by: 14 days	[33]
9	4Alg:1PAM hydrogel	65 g/g in tap water at room temperature	Retains 30% of its water content after 5 days at 30 °C and 30% relative humidity	[34]
10	CMC-PNIPAM semi-IPN hydrogel	Maximum Swelling Ratio: 170.25 g·g^−1^ (dry gel) at 25 °C; Swelling Rate: 2.57 g·g^−1^·min^−1^	Soil mixed with 2 wt% dry hydrogel retained 13.08 wt% water after 30 days at 25 °C—4.4 times higher than control soil	[35]
11	SA-g-P(AA-co-AMPS)/GO Hydrogel Composite	Maximum absorption in distilled water: 862.3 g/g; Maximum absorption in 0.9% NaCl solution: 164.7 g/g	Water retention rate after 6 h at 60 °C: 80%; Water retention rate after 6 h at 100 °C: 51%	[36]

**Table 2:** Bio-gels as heavy metal ion adsorbents.

No.	Gel Type	Experimental Parameters	Heavy Metal Ion Adsorption Performance	References
1	Cross-linked chitosan Gel beads (BGC)	pH: 5.0; Temperature: 25 °C; Initial Concentration: 125 mg/L	Target Metals: Co^2+^, Cd^2+^, Pb^2+^ Maximum Adsorption Capacity (mg/g): Co^2+^ (73.98), Cd^2+^ (68.52), Pb^2+^ (78.67)	[16]
2	Radiation-cross-linked chitosan-polyacrylamide hydrogel (Chi-co-PAAm)	pH: 9.0; Initial Concentration: 10–100 mg/L; Adsorbent Dosage: 20 mg/20 mL (1 g/L)	Target Metals: Cu^2+^, Cr^6+^, Ni^2+^, Pb^2+^, Co^2+^, Zn^2+^ Adsorption Capacity Order: Zn^2+^ > Cr^6+^ > Pb^2+^ > Cu^2+^ > Co^2+^ > Ni^2+^ Maximum Adsorption Capacity (mg/g): Zn^2+^ (333.3), Pb^2+^ (66.7)	[22]
3	Mixed anionic (NIPAM-co-acrylic acid) and cationic (NIPAM-co-Chitosan) gels	Temperature: 10 °C; Initial Concentration: 10 mM (≈2072 mg/L for Pb^2+^); Adsorbent Dosage: 1 g/20 mL (50 g/L)	Target Metals: Pb^2+^, Fe^2+^, Ni^2+^ Adsorption Order: Pb^2+^ > Fe^2+^ > Ni^2+^ Maximum Adsorption Capacity (mg/g): Pb^2+^ (93.15)	[23]
4	Ternary composite hydrogel beads (SCP@PEI), composed of sodium alginate (SA), cellulose nanofibers (CNF), and polyethyleneimine (PEI)	pH: 3.0; Temperature: 25 °C; Initial Cr(VI) Concentration: 1–10 mmol/L (Isotherm study); Adsorbent Dosage: 0.4 g/L	Target Metal: Cr(VI) Maximum Adsorption Capacity: 6.49 mmol/g (approx. 337 mg/g)	[24]
5	Sodium alginate gel-based adsorbents (including gel beads, capsules, and gel-coated cotton)	pH: 5.0; Temperature: 25 °C; Initial Pb^2+^ Concentration: 200 mg/L (200 mg dm^−3^); Adsorbent Dosage: 0.1 g/L (based on dry sodium alginate weight)	Target Metal: Pb^2+^ Adsorption Capacity (mg/g): Gel Beads: 450 Gel Capsules: 1560 Gel-Coated Cotton: 521 (using 0.5% sodium alginate)	[12]
6	Polyacrylamide/Modified silica composite hydrogel (PAM/SiO_2_-NH_2_)	pH: 5.0; Temperature: 25 °C; Initial Metal Concentration: 5 mmol/L (0.005 mol/L)	Target Metals: Cu(II), Cd(II), Pb(II) Adsorption Order: Pb(II) > Cu(II) > Cd(II)	[25]
7	Hydrolyzed polyacrylamide–chitosan composite gel beads (HPAM-Chitosan)	pH: 4.2; Temperature: 30 °C; Initial Metal Concentration: 1 mmol/L; Adsorbent Dosage: 0.2 g/L	Target Metals: Cu^2+^, Pb^2+^, Hg^2+^ Adsorption Order: Pb^2+^ > Cu^2+^ > Hg^2+^ Equilibrium Adsorption Capacity (mmol/g): Pb^2+^ (1.69), Cu^2+^ (0.69), Hg^2+^ (0.51)	[21]
8	PAM/PAA/PDMTM gel	pH: 5.0; Temperature: 25 °C; Initial Concentration: 50 mg/L; Adsorbent Dosage:1 g/L	Target Metals: Cu^2+^, Cd^2+^, Pb^2+^ Adsorption Order: Pb^2+^ > Cu^2+^ > Cd^2+^ Maximum Adsorption Capacity (mg/g): Cu^2+^ (92.33), Pb^2+^ (200.97), Cd^2+^ (110.08)	[53]
9	SCS-gel (Sulfonated Corn Stalk-based Double-Network Hydrogel)	pH Range: 2.0–6.0 (for Pb^2+^), 3.0–6.0 (for Cu^2+^); Temperature: °C; Initial Concentration (Batch): 100–300 mg/L	Target Metals: Pb^2+^, Cu^2+^, Mn^2+^, Zn^2+^, Al^3+^ Adsorption Order: Pb^2+^ > Cu^2+^ > Al^3+^ > Zn^2+^ > Mn^2+^ Max Adsorption Capacity: Pb^2+^ = 111.6 mg/g; Cu^2+^ = 370.2 mg/g	[54]
10	β-Cyclodextrin-based hydrogel (CAM)	pH: 5.0; Initial Concentration: 25, 50, 100, 150 mg/L; Adsorbent Dosage: 0.01–0.2 g/L	Target Metals: Cd^2+^, Pb^2+^, Cu^2+^ Adsorption Order: Pb^2+^ > Cu^2+^ > Cd^2+^ Max Adsorption Capacity (mg/g): Pb^2+^ (210.6), Cu^2+^ (116.41), Cd^2+^ (98.88)	[55]

**Table 3:** Advantages, disadvantages, and applicability of four bio-gel remediation methods in environmental fields.

No.	Methods	Introduction	Advantages	Disadvantages	Applicability	References
1	Heavy metal ion adsorption	Utilizing functional groups (e.g., carboxyl, amino, and hydroxyl groups) within the three-dimensional network structure of bio-gels, heavy metal ions in water or soil can be immobilized and removed through mechanisms such as ion exchange, coordination, and electrostatic adsorption	1. High adsorption capacity with tunable selectivity 2. Broad raw material sources and low cost 3. Excellent biocompatibility and biodegradability 4. Ease of functionalization and performance optimization	1. Requirement for post-saturation treatment with potential secondary pollution risk 2. Significant influence of environmental conditions on adsorption performance 3. Limited mechanical strength leading to structural instability in practical flow systems 4. Generally restricted selectivity toward specific metal ions	1. Industrial wastewater treatment 2. In situ remediation of contaminated water bodies/sediments 3. Immobilization of heavy metals in lightly contaminated soils	[48,49,50,52]
2	Microbial immobilization	Functional microorganisms (e.g., degrading bacteria, nitrifying bacteria) are encapsulated or immobilized within the porous network of bio-gels, which act as a “microbial shelter.” This strategy significantly enhances microbial activity, stability, and retention capacity in contaminated areas, enabling efficient and sustained bioremediation.	1. Shielding microorganisms from toxic substances and environmental stresses 2. Enhancing microbial concentration and activity for improved remediation efficiency 3. Serving as an additional carbon source to support microbial metabolism 4. Enabling reusability and suitability for bioreactor systems	1. Potential diffusion limitations for substrates and products 2. Risk of microbial damage during immobilization procedures 3. Trade-off between long-term stability and biodegradability 4. Higher cost and technical requirements compared to free-cell application	1. Bioaugmentation remediation of recalcitrant organic pollutants (e.g., petroleum hydrocarbons, pesticides) 2. Nitrogen and phosphorus removal from water bodies (nitrification/denitrification) 3. Microbial remediation in high-toxicity environments, providing a survival microenvironment for functional bacteria	[7,56,57]
3	Soil improvement and ecological restoration	Utilizing the superabsorbent and water-retaining capabilities of bio-gels (particularly cellulose-based gels, among others) as soil amendments to improve soil structure, provide sustained moisture for plant growth, and alleviate drought stress	1. Significantly improves soil water retention capacity, reducing water evaporation and irrigation requirements 2. Improves soil physical structure, increases porosity, and promotes the formation of aggregate structure 3. Promotes seed germination and plant growth, improving survival rates 4. Raw materials are biodegradable, eventually integrating into the soil without leaving residues	1. Long-term use may affect soil permeability 2. Its effectiveness is significantly influenced by soil type and climatic conditions 3. The degradation cycle of some synthetic-based gels is uncontrollable 4. Cost remains a consideration in large-scale applications	1. Agricultural and ecological restoration in arid and semi-arid regions 2. Desertification control and sandy soil improvement 3. Vegetation restoration under challenging site conditions such as slope greening and mine reclamation	[3,4]
4	Controlled release of active agents	Loading pesticides, fertilizers, and other agricultural chemicals into the network of bio-gels, and utilizing their environmental responsiveness (e.g., to pH, enzymes, moisture) to achieve slow and controlled release of active ingredients through diffusion, swelling, or degradation	1. Improve utilization efficiency, reducing the dosage and loss of chemical fertilizers and pesticides 2. Reduce environmental pollution, lowering the risk of contamination of groundwater and soil 3. Prolong the effective duration, reducing application frequency and labor costs 4. Strong controllability, enabling precise regulation of release behavior through the design of gel composition and structure	1. The manufacturing process is relatively complex, with costs higher than those of traditional formulations 2. A balance between loading capacity and release efficiency requires optimization 3. Prediction and control of release kinetics still require in-depth research 4. Market promotion and farmer acceptance require a gradual process	1. Controlled release of chemical fertilizers and pesticides in precision and sustainable agriculture 2. Horticulture and silviculture requiring long-term nutrient supply 3. Agricultural activities in environmentally sensitive areas, aiming to reduce non-point source pollution	[61,62]

## Data Availability

Data will be made available on request.

## References

[1] Bao T., Wang P., Hu B., Jin Q., Zheng T., Li D. (2024). Adsorption and distribution of heavy metals in aquatic environments: The role of colloids and effects of environmental factors. J. Hazard. Mater..

[2] Waqas W., Yuan Y., Ali S., Zhang M., Shafiq M., Ali W., Chen Y., Xiang Z., Chen R., Ikhwanuddin M. (2024). Toxic effects of heavy metals on crustaceans and associated health risks in humans: A review. Environ. Chem. Lett..

[3] Tariq Z., Iqbal D.N., Rizwan M., Ahmad M., Faheem M., Ahmed M. (2023). Significance of biopolymer-based hydrogels and their applications in agriculture: A review in perspective of synthesis and their degree of swelling for water holding. RSC Adv..

[4] Fang S., Zhong Y., Wu J., Xie Y., Cai L., Li M., Cao J., Zhao H., Dong B. (2024). A comparative analysis of the water retention properties of hydrogels prepared from melon and orange peels in soils. Gels.

[5] Xie Y., Zhong Y., Wu J., Fang S., Cai L., Li M., Cao J., Zhao H., Dong B. (2024). “From Waste to Wonder”: Comparative Evaluation of Chinese Cabbage Waste and Banana Peel Derived Hydrogels on Soil Water Retention Performance. Gels.

[6] Guo S., Su K., Yang H., Zheng W., Zhang Z., Ang S., Zhang K., Wu P. (2022). Novel natural glycyrrhetinic acid-derived super metal gel and its highly selective dyes removal. Gels.

[7] Zheng L., Seidi F., Wu H., Huang Y., Wu W., Xiao H. (2024). Low swelling Alginate/Lignin network gels with redox responsiveness for sustained release of agricultural fungicide and Pb^2+^ complexation. Eur. Polym. J..

[8] Kim D., Kim D.H., Cha J.E., Park S., Lee S.H. (2025). Metal-Ion-Free Preparation of κ-Carrageenan/Cellulose Hydrogel Beads Using an Ionic Liquid Mixture for Effective Cationic Dye Removal. Gels.

[9] Senesi N., Loffredo E. (2008). The fate of anthropogenic organic pollutants in soil: Adsorption/desorption of pesticides possessing endocrine disruptor activity by natural organic matter (humic substances). Rev. Cienc. Suelo Nutr. Veg..

[10] Xu L., Chen M., Cui Q., Wang C., Zhang M., Zheng L., Li S., Zhang H., Liang G. (2023). Ultra-clean ternary Au/Ag/AgCl nanoclusters favoring cryogenic temperature-boosted broadband SERS ultrasensitive detection. Opt. Express.

[11] Mohammad R.E.A., Veerasingam S., Suresh G., Rajendran S., Sadasivuni K.K., Ghani S., Al-Khayat F. (2025). Tackling Environmental Radionuclides Contamination: A Systematic Review of Chemical, Biological, and Physical Remediation Strategies. Chem. Eng. J. Adv..

[12] Park H.G., Chae M.Y. (2004). Novel type of alginate gel-based adsorbents for heavy metal removal. J. Chem. Technol. Biotechnol..

[13] Petre M., Zarnea G., Adrian P., Gheorghiu E. (1999). Biodegradation and bioconversion of cellulose wastes using bacterial and fungal cells immobilized in radiopolymerized hydrogels. Resour. Conserv. Recycl..

[14] Thakur S., Arotiba O. (2018). Synthesis, characterization and adsorption studies of an acrylic acid-grafted sodium alginate-based TiO_2_ hydrogel nanocomposite. Adsorpt. Sci. Technol..

[15] Mishra A., Nath A., Pande P.P., Shankar R. (2021). Treatment of gray wastewater and heavy metal removal from aqueous medium using hydrogels based on novel crosslinkers. Appl. Polym. Sci..

[16] Lemrabet E., Jaafari K., Benkhouja K., Touaiher M., Bakasse M., Sahraoui B., Touhtouh S., Soufiáne A. (2013). Removal of heavy-metal ion by adsorption on chitosan gel beads. J. Optoelectron. Adv. Mater..

[17] Marciano J.S., Ferreira R.R., de Souza A.G., Barbosa R.F., de Moura Junior A.J., Rosa D.S. (2021). Biodegradable gelatin composite hydrogels filled with cellulose for chromium (VI) adsorption from contaminated water. Int. J. Biol. Macromol..

[18] Zhang Y., Li H., Zhang Z., Li X., Cheng K., Chen G., Yang Y., Wang S., Liu T. (2024). Efficient nitrogen removal from low-C/N ratio wastewater by a new gel microsphere with nitrate-reducing Fe (II)-oxidizing bacteria. ACS EST Water.

[19] Blebea N.M., Pușcașu C., Vlad R.A., Hancu G. (2025). Chitosan-based gel development: Extraction, gelation mechanisms, and biomedical applications. Gels.

[20] Sun R., Gao S., Zhang K., Cheng W.T., Hu G. (2024). Recent advances in alginate-based composite gel spheres for removal of heavy metals. Int. J. Biol. Macromol..

[21] Cao J., Tan Y., Che Y., Xin H. (2010). Novel complex gel beads composed of hydrolyzed polyacrylamide and chitosan: An effective adsorbent for the removal of heavy metal from aqueous solution. Bioresour. Technol..

[22] Puspitasari T., Oktaviani, Pangerteni D.S., Nurfilah E., Darwis D. (2015). Study of Metal Ions Removal from Aqueous Solution by Using Radiation Crosslinked Chitosan-co-Poly (Acrylamide)-Based Adsorbent. Macromol. Symp..

[23] Ningrum E.O., Suprapto S., Altway S., Triastuti W.E., Hamzah A., Surono A., Taji L.S., Ardiansyah E. (2024). Effect of Synthesis Temperature on Adsorbent Performance of Blending Anionic and Cationic Gels in Divalent Metal Ions Adsorption. Indones. J. Chem..

[24] Feng Y., Zhang X., Qiu Y., Song L., Liu F. (2021). Efficient Removal Characteristics and Mechanism of Cr(VI) in water by Ternary Composite Hydrogel Beads. Ion Exch. Adsorpt..

[25] Wang J., Li X. (2015). Synthesis of polyacrylamide/modified silica composite hydrogels for synergistic complexation of heavy metal ions. Desalin. Water Treat.

[26] Wang S., Wang Y., Xiao Z., Xie Z. (2022). Recent Progress in Preparation and Application of Bio-based Hydrogels. Chem. Ind. For. Prod..

[27] Guo H., Jiao T., Zhang Q., Guo W., Peng Q., Yan X. (2015). Preparation of graphene oxide-based hydrogels as efficient dye adsorbents for wastewater treatment. Nanoscale Res. Lett..

[28] Wu T., Sawut A., Simayi R. (2025). Preparation and Heavy Metal Adsorption Performance of 2-Aminopyridine-Modified Sodium Alginate/Polyacrylic Acid Hydrogel. Gels.

[29] Montesano F.F., Parente A., Santamaria P., Sannino A., Serio F. (2015). Biodegradable superabsorbent hydrogel increaseswater retention properties of growing media and plant growth. Agric. Agric. Sci. Procedia.

[30] He L., Guo Y., Wang Z., Hou K., Wei L., Zhang J. (2025). Characterization and Application of Cellulose-Humic Acid-Based Composite Superabsorbent Hydrogel. Polym. Sci. Ser. B.

[31] Liu C., Tang M., Zhang F., Lei F., Li P., Wang K., Zeng H., Jiang J. (2022). Facile access to gleditsia microphylla galactomannan hydrogel with rapid self-repair capacity and multicyclic water-retaining performance of sandy soil. Polymers.

[32] Ghobashy M.M., Amin M.A., Mustafa A.E., El-Diehy M.A., El-Damhougy B.K., Nady N. (2024). Synthesis and application of a multifunctional poly (vinyl pyrrolidone)-based superabsorbent hydrogel for controlled fertilizer release and enhanced water retention in drought-stressed Pisum sativum plants. Sci. Rep..

[33] Chen X., Yang T., Cai X., Liu Y., Huang C., He J., Tian D., Yang G., Shen F., Zhang Y. (2024). Eco-friendly hydrogel based on locust bean gum for water retaining in sandy soil. International Int. J. Biol. Macromol..

[34] Pettinelli N., Sabando C., Rodríguez-Llamazares S., Bouza R., Castano J., Valverde J.C., Rubilar R., Frizzo M., Recio-Sánchez G. (2024). Sodium alginate-g-polyacrylamide hydrogel for water retention and plant growth promotion in water-deficient soils. Ind. Crops Prod..

[35] Chen G., Ma F., Li J., Yang P., Wang Y., Li Z., Meng Y. (2024). Preparation of CMC-poly(N-isopropylacrylamide) semi-interpenetrating hydrogel with temperature-sensitivity for water retention. Int. J. Biol. Macromol..

[36] Zhu L., Liu Y., Wang F., He T., Tang Y., Yang J. (2018). Preparation and the swelling properties of sodium alginate graft poly(acrylic acid-co-2-acrylamide-2-methyl propane sulfonic acid)/graphene oxide hydrogel composite. Adv. Polym. Technol..

[37] Li H., Zhao L., Chen C., Zhu H., Mao X. (2025). Immobilizing polycyclic aromatic hydrocarbon degrading bacteria with gel microspheres for bioaugmentation remediation of soil in a surfactant-enhanced slurry bioreactor. J. Clean. Prod..

[38] Jin X., Xu H., Mao Z., Feng X., Zhong Y. (2025). Polysaccharide Hydrogels Based on Cellulose and Chitosan for Drug Sustained-Release Applications. Appl. Sci..

[39] Dubey S., Sharma A., Kaur K., Pal K., Mohsin M.E.A., Mousa S. (2025). Novel chemical integration of biodegradable energy storage materials utilizations for sustainable environment. Mol. Struct..

[40] Yang S., Liu Z., Pan Y., Guan J., Yang P., Asel M. (2023). A review of research progress on the performance of intelligent polymer gel. Molecules.

[41] Liu Z., Faraj Y., Ju X.J., Wang W., Xie R., Chu L.Y. (2018). 2018. Nanocomposite smart hydrogels with improved responsiveness and mechanical properties: A mini review. J. Polym. Sci. Pol. Phys..

[42] Kumari S., Avais M., Chattopadhyay S. (2023). Microgels as smart polymer colloids for sensing and environmental remediation. ACS Appl. Polym. Mater..

[43] Stetsyshyn Y., Ohar H., Budkowski A., Lazzara G. (2025). Molecular design and role of the dynamic hydrogen bonds and hydrophobic interactions in temperature-switchable polymers: From understanding to applications. Polymers.

[44] Ma M., He Z., Zhou S., Liu X., Zhao M., Wang X., Liu H., Hao A. (2022). A β-cyclodextrin/graphene oxide hybrid gel with smart responsiveness. J. Incl. Phenom. Macrocycl. Chem..

[45] Guan W.L., Adam K.M., Qiu M., Zhang Y.M., Yao H., Wei T.B., Lin Q. (2020). Research progress of redox-responsive supramolecular gel. Supramol. Chem..

[46] Mondal B., Bairagi D., Nandi N., Hansda B., Das K.S., Edwards-Gayle C.J., Castelletto V., Castelletto I.W., Banerjee A. (2020). Peptide-based gel in environmental remediation: Removal of toxic organic dyes and hazardous Pb^2+^ and Cd^2+^ ions from wastewater and oil spill recovery. Langmuir.

[47] Mazzeo L., Rosa D., Bavasso I., Di Palma L. (2021). Entrapped zinc oxide and titania nanoparticles in calcium alginate beads for the removal of Methylene Blue (MB). Adsorption properties and photocatalytic stability. Chem. Eng. Trans..

[48] Chen M., Long A., Zhang W., Wang Z., Xiao X., Gao Y., Zhou L., Li Y., Wang J., Sun S. (2025). Recent advances in alginate-based hydrogels for the adsorption–desorption of heavy metal ions from water: A review. Sep. Purif. Technol..

[49] Sun J., Zhang Z., Yu M., Xu W., Bai G., Xiang Y., Li M. (2025). Chitosan Modified Diatomite Cross-Linked with Sodium Alginate and Polyethyleneimine Composite Gel Particles for Efficient Removal of Copper and Lead Ions. Polym. Environ..

[50] Song Y., Gotoh T., Nakai S. (2024). Simultaneous Removal of Anionic and Cationic Species by Ionic Hydrogels via Positively Charged Complex: Adsorption Mechanism of Arsenic (V) and Chromium (III) in Highly Acidic System. ACS EST Water.

[51] Hayeeye F., Yu Q.J., Sattar M., Chinpa W., Sirichote O. (2018). Adsorption of Pb2+ ions from aqueous solutions by gelatin/activated carbon composite bead form. Adsorpt. Sci. Technol..

[52] Hui B., Zhang Y., Ye L. (2015). Structure of PVA/gelatin hydrogel beads and adsorption mechanism for advanced Pb (II) removal. Ind. Eng. Chem..

[53] Niu H.Y., Li J.C., Li J.S., Yi C., Niu C.G. (2023). Preparation, properties and applications of porous hydrogels containing thiol groups for heavy metal removal. Environ. Chem. Eng..

[54] Feng Z., Li J., Chen N., Feng C. (2025). Sulfonated corn stalk enhanced hydrogel adsorption for heavy metal from metal mine gallery effluent. Sep. Purif. Technol..

[55] Huang Z., Wu Q., Liu S., Zhang B. (2013). A novel biodegradable β-cyclodextrin-based hydrogel for the removal of heavy metal ions. Carbohydr. Polym..

[56] Borin G.P., de Melo R.R., Crespim E., Sato H.H., Contesini F.J., Thakur V., Thakur M. (2018). An overview on polymer gels applied to enzyme and cell immobilization. Polymer Gels: Science and Fundamentals.

[57] Guo X., Jiang G., Fu S., Hu Z., Di J., Li Y. (2021). Treatment of Agate Dyeing Wastewater Using an Immobilized Gel Mixture with Nano-Fe_3_O_4_ Sulfate-Reducing Bacteria. Renew. Mater..

[58] Muhauwiss F.M., Hussein A.H., Hussein M.A. (2024). Shear strength durability investigation for gypseous soil enhanced by pectin biopolymer. Tikrit J. Eng. Sci..

[59] Qin C.C., Abdalkarim S.Y.H., Zhou Y., Yu H.Y., He X. (2022). Ultrahigh water-retention cellulose hydrogels as soil amendments for early seed germination under harsh conditions. J. Clean. Prod..

[60] Wei J., Yang H., Cao H., Tan T. (2016). Using polyaspartic acid hydro-gel as water retaining agent and its effect on plants under drought stress. Saudi J. Biol. Sci..

[61] Liu H., Gao Z., Cai T., Zhang Y., Miao Q., Cui Z. (2024). Chitosan gels in the nutrient controlled release: A review. J. Plant Nutr..

[62] Muharam S., Fitri A., Yuningsih L.M., Putri Y.M.T.A., Rahmawati I. (2020). Synthesis and characterization of controlled-release urea fertilizer from superabsorbent hydrogels. Indones. J. Chem..

[63] Wei X., Bao X., Yu L., Liu H., Lu K., Chen L., Bai L., Zhou X., Li Z., Li W. (2020). Correlation between gel strength of starch-based hydrogel and slow release behavior of its embedded urea. Polym. Environ..

[64] Ungureanu E., Mikhailidi A., Tofanica B.M., Fortună M.E., Rotaru R., Ungureanu O.C., Samuil C., Popa V.I. (2025). Sustainable Gels from Polysaccharides in Agriculture. Polysaccharides.

[65] Kabir M.H., Ahmed K., Furukawa H. (2017). A low cost sensor based agriculture monitoring system using polymeric hydrogel. Electrochem. Soc..

[66] Zhou X., Zhang P., Zhao F., Yu G. (2020). Super moisture absorbent gels for sustainable agriculture via atmospheric water irrigation. ACS Mater. Lett..

[67] Zhang Z., Sun L., Huo X., Liu X., Pan X. (2024). Rheological properties and gas channeling plugging ability in CO_2_ flooding of a hydrophobic nanoparticle-enhanced smart gel system constructed with wormlike micelles. Chem. Eng. Res. Des..

[68] Yang J.B., Sun J.S., Bai Y.R., Lv K.H., Wang Z.Y., Xu C.Y., Dai L.Y., Wang R. (2022). Review of the application of environmentally responsive gels in drilling and oil recovery engineering: Synthetic materials, mechanism, and application prospect. J. Petrol. Sci. Eng..

[69] Zhao Y., Yang Q., Khalaf A.H., Lin B., Tang J. (2025). Long-Term Anti-Corrosion Performance of Ultra-High Content Inhibitor Loaded Gel-Epoxy Solid Inhibitor with Temperature-Responisve Effect. Appl. Sci..

[70] Yin P., Shi F., Luo M., Wu J., Zhao B., Zhang C., Shen Y., Chen Y. (2024). Preparation and Characterization of Responsive Cellulose-Based Gel Microspheres for Enhanced Oil Recovery. Gels.

[71] Geng S., Zhang H., Zhang Y., Liu L., Yu S., Lan X., Gao Y., Ling Z., Zhang Y., Li X. (2024). Puerarin hydrogel: Design and applications in biomedical engineering. J. Drug Delivery Sci. Technol..

[72] Sharifi E., Chehelgerdi M., Fatahian-Kelishadrokhi A., Yazdani-Nafchi F., Ashrafi-Dehkordi K. (2021). Comparison of therapeutic effects of encapsulated Mesenchymal stem cells in Aloe vera gel and Chitosan-based gel in healing of grade-II burn injuries. Regen. Ther..

[73] Zhang Y., Wu B.M. (2023). Current advances in stimuli-responsive hydrogels as smart drug delivery carriers. Gels.

[74] Grove T.Z., Osuji C.O., Forster J.D., Dufresne E.R., Regan L. (2010). Stimuli-responsive smart gels realized via modular protein design. J. Am. Chem. Soc..

[75] Li J., He Z., Yang Y., Lan Y., Dong Z., Zou M., Li W., Zeng D., Yang Y., Chen X. (2025). ARF Biogel Enriched with Osteogenic Differentiation Regulators Guides BMSCs Osteogenic Differentiation toote Bone Tissue Regeneration after Osteonecrosis of the femoral head through Vascular Remodeling and Synchronized Regulation of Inflammatory Response. Research Square.

